# The proteasome regulator Rpn4 controls antifungal drug tolerance by coupling protein homeostasis with metabolic responses to drug stress

**DOI:** 10.1371/journal.ppat.1011338

**Published:** 2023-04-19

**Authors:** Ka Pui Sharon Yau, Harshini Weerasinghe, Francios A. B. Olivier, Tricia L. Lo, David R. Powell, Barbara Koch, Traude H. Beilharz, Ana Traven

**Affiliations:** 1 Infection Program, Department of Biochemistry & Molecular Biology, Biomedicine Discovery Institute, Monash University, Clayton, Victoria, Australia; 2 Centre to Impact AMR, Monash University, Clayton, Victoria, Australia; 3 Bioinformatics Platform, Monash University, Clayton, Victoria, Australia; 4 Development and Stem Cells Program, Department of Biochemistry & Molecular Biology, Biomedicine Discovery Institute, Monash University, Clayton, Victoria, Australia; University of Georgia, UNITED STATES

## Abstract

Fungal pathogens overcome antifungal drug therapy by classic resistance mechanisms, such as increased efflux or changes to the drug target. However, even when a fungal strain is susceptible, trailing or persistent microbial growth in the presence of an antifungal drug can contribute to therapeutic failure. This trailing growth is caused by adaptive physiological changes that enable the growth of a subpopulation of fungal cells in high drug concentrations, in what is described as drug tolerance. Mechanistically, antifungal drug tolerance is incompletely understood. Here we report that the transcriptional activator Rpn4 is important for drug tolerance in the human fungal pathogen *Candida albicans*. Deletion of *RPN4* eliminates tolerance to the commonly used antifungal drug fluconazole. We defined the mechanism and show that Rpn4 controls fluconazole tolerance via two target pathways. First, Rpn4 activates proteasome gene expression, which enables sufficient proteasome capacity to overcome fluconazole-induced proteotoxicity and the accumulation of ubiquitinated proteins targeted for degradation. Consistently, inhibition of the proteasome with MG132 eliminates fluconazole tolerance and resistance, and phenocopies the *rpn4Δ/Δ* mutant for loss of tolerance. Second, Rpn4 is required for wild type expression of the genes required for the synthesis of the membrane lipid ergosterol. Our data indicates that this function of Rpn4 is required for mitigating the inhibition of ergosterol biosynthesis by fluconazole. Based on our findings, we propose that Rpn4 is a central hub for fluconazole tolerance in *C*. *albicans* by coupling the regulation of protein homeostasis (proteostasis) and lipid metabolism to overcome drug-induced proteotoxicity and membrane stress.

## Author summary

Microbial pathogens use a number of mechanisms to overcome antimicrobial therapy. Understanding these mechanisms should provide the knowledge base for designing improved treatments. Here we studied how the human fungal pathogen *Candida albicans* overcomes growth inhibition by the antifungal drug fluconazole. Our focus was on the process of drug tolerance, which drives trailing microbial growth in the presence of antifungal drugs. We identified the transcriptional activator Rpn4 as an important regulator of fluconazole tolerance in *C*. *albicans*. Rpn4 regulates the expression of genes encoding proteasome subunits, and our data suggests that Rpn4-dependent regulation of the proteasome contributes to fluconazole tolerance by maintaining protein homeostasis in response to drug stress. Rpn4 is also required for normal expression of the genes required for the synthesis of the membrane lipid ergosterol, which could further contribute to overcoming fluconazole’s inhibition of this metabolic pathway. In conclusion, Rpn4 contributes to fluconazole tolerance by regulating the cellular adaptation mechanisms that ensure protein and lipid homeostasis in response to drug-induced stress. As such, inactivation of Rpn4 or inhibition of the proteasome eliminate drug tolerance and increase fluconazole susceptibility in *C*. *albicans*.

## Introduction

The yeast *Candida albicans* causes life-threatening infections in patients with reduced immune function [1]. Treating these infections remains challenging, as there are only a handful of clinically useful antifungal drugs [2]. New antifungal drugs are being developed [3], but it is also important to understand how fungi overcome current antifungal agents so that improved treatment strategies could be devised [4]. In this report, we focus on understanding how *C*. *albicans* overcomes fluconazole, an azole drug that is widely used for the treatment of fungal infections [2].

Azoles inhibit Erg11, a cytochrome P450 enzyme in the biosynthetic pathway of the fungal lipid ergosterol. Inhibition of Erg11 causes membrane stress because ergosterol is depleted and toxic sterol metabolites accumulate. The azoles drugs are active against a range of fungal pathogens, however the evolution of drug resistance can cause treatments to fail [4]. Azole resistance is relatively well understood. It involves classic drug-resistance mechanisms: activation of drug efflux, increased levels of the drug target (in this case Erg11) and mutations in the target that reduce drug binding, reviewed in [4]. *C*. *albicans* can further overcome azole-induced growth inhibition by a different set of mechanisms that have collectively been termed “*drug tolerance*” [4–7]. Tolerance mechanisms reflect a heterogenous behaviour of cell populations, whereby some (but not all) cells are able to overcome drug-induced growth arrest and then divide at high drug concentrations [4,5,8]. As such, tolerance describes trailing or persistent growth in the presence of an antifungal drug, which could contribute to poor patient outcomes [4,5,7,8].

Although it is likely that there is some mechanistic overlap between azole resistance and tolerance, these two responses are considered to be distinct because they can be uncoupled in response to chemical inhibitors or mutations in certain cellular pathways [4]. For example, inhibitors of stress signalling decrease fluconazole tolerance by reducing the fraction of the cell population that can grow in high drug concentrations. However, drug resistance remains the same in the presence of these inhibitors, since the minimal inhibitory drug concentration (MIC) for the fungal cell population as a whole is unchanged [4,8].

The mechanisms responsible for azole tolerance are incompletely defined, although some patterns are emerging. For instance, tolerance of *C*. *albicans* to fluconazole is related to the ability of some cells in the population to limit the intracellular drug concentration [8]. Another proposed mechanism of azole tolerance is linked to changes in membrane composition, whereby increased sphingolipid biosynthesis can compensate for reduced ergosterol biosynthesis in the presence of azoles [8–10]. Additionally, several stress responders, such as the chaperone Hsp90, calcineurin, PKC (protein kinase C) and TOR (target of rapamycin) have been reported to modulate azole tolerance [8,11–14], reviewed in [4]. Identifying the full spectrum of azole tolerance mechanisms should build the knowledge base towards designing improved treatments for *C*. *albicans* infections.

In this study we reveal an important role for the proteasome and its regulator, the transcription factor Rpn4, in fluconazole tolerance by *C*. *albicans*. We show that fluconazole tolerance depends on the cell’s ability to overcome proteotoxic stress and the accumulation of misfolded proteins caused by fluconazole. Rpn4-dependent transcription of proteasome genes provides sufficient proteasome capacity to ensure that protein homeostasis (i.e. proteostasis) is maintained in drug. Rpn4 further contributes to the expression of ergosterol and heme biosynthesis genes, which are needed in response to inhibition of ergosterol biosynthesis by fluconazole. Collectively, our results show that Rpn4 coordinates the cellular response to fluconazole by mitigating proteotoxicity and membrane stress. Our study identifies Rpn4 and the proteasome as targets for disabling fluconazole tolerance to improve drug efficacy.

## Results

### Roles of Rpn4 in stress responses, morphogenesis and immune interactions of *C*. *albicans*

The transcriptional activator Rpn4 is best characterised in the model yeast *Saccharomyces cerevisiae*, where it is required for activation of the proteasome genes [15]. Its roles in *C*. *albicans* have not been studied in any detail. The *C*. *albicans RPN4* gene is activated in the core stress response [16]. Therefore, we started by asking if the *rpn4Δ/Δ* mutant has any stress susceptibility phenotypes. In plate assays, *rpn4Δ/Δ* displayed wild type susceptibility to most of the tested stressors including cell wall, membrane, osmotic and respiratory stress (S1A Fig). The exceptions were as follows. The mutant displayed mild susceptibility to hydrogen peroxide and DMSO at 30°C and tunicamycin at 37°C (S1A Fig). The strongest phenotype of *rpn4Δ/Δ* was its susceptibility to fluconazole at 37°C (S1A Fig). The mutant also had a moderate growth defect with a ~15% slower growth rate in liquid medium (S1B–S1D Fig). The growth and stress phenotypes were generally consistent between two independent deletion clones (x and y), and were complemented when the wild type *RPN4* gene was re-introduced into the mutants (S1A–S1D Fig).

*C*. *albicans* grows in distinct cellular morphologies depending on environmental conditions [17]. Cultures of *rpn4Δ/Δ* cells displayed a higher proportion of filamentous morphologies (hyphae and pseudohyphae) under conditions in which wild type cells were predominantly in yeast form (S2A and S2B Fig). The mutant formed normal hyphae *in vitro* in conditions that trigger hyphal formation (S2C Fig). The mutant also formed normal hyphae upon phagocytosis by macrophages (S2D Fig, S1 Movie). These data suggest that Rpn4 is required for maintaining yeast morphology, while it does not have a major role in hyphal growth.

Hyphal formation enables the escape of *C*. *albicans* from macrophages by promoting host cell lysis via multiple mechanisms [18–20]. In addition to hyphae-induced lysis, macrophages further die due to metabolic stress caused by *C*. *albicans* infection, which is triggered by rapid depletion of glucose upon fungal growth [21]. Macrophage infection rates were similar for the *rpn4Δ/Δ* mutant and wild type controls (S3A Fig), but macrophages infected with *rpn4Δ/Δ* displayed slower cell death (S3B Fig). Consistent with this, the *rpn4Δ/Δ* mutant depleted glucose more slowly (S3C Fig).

*C*. *albicans*-infected macrophages become dependent on glucose due to their increased glycolysis [21]. The shift of macrophages to increased glycolysis can be assessed by measuring transcriptional signatures, such as the levels of glycolytic enzymes and the glucose importer *Glut1*. Infection of macrophages with the *rpn4Δ/Δ* mutant induced these transcriptional signatures of increased macrophage glycolysis (S3D Fig). Therefore, the slower cell death of *rpn4Δ/Δ*-infected macrophages is not explained by changes to immune cell metabolism. Rather, our data indicates that the slower growth and metabolic changes in the *rpn4Δ/Δ* mutant cause slower glucose depletion by *C*. *albicans*, which in turn prolongs macrophage viability.

Taken together, our phenotypic characterisation shows that Rpn4 contributes to drug responses, morphogenesis and immune cell interaction of *C*. *albicans*. The strongest phenotype of *rpn4Δ/Δ* that we detected was its hyper-susceptibility to fluconazole (S1A Fig).

### Rpn4 has a prominent function in fluconazole tolerance and a more modest role in resistance

Our *rpn4Δ/Δ* mutant clones were obtained from the Homann transcription factor mutant library [22]. Previous screens of that library are consistent with our data in that they also reported fluconazole susceptibility for *rpn4Δ/Δ* [22,23]. However, detailed analyses of this phenotype have not been performed. As discussed in the Introduction, *C*. *albicans* overcomes fluconazole by a complex set of processes that include two distinct components: resistance and tolerance [4]. Therefore, to precisely decipher the function of Rpn4 in fluconazole susceptibility, we utilised disk diffusion assays which allow for the determination of drug resistance (based on the radius of the zone of inhibition or “RAD”) as well as drug tolerance (based on fungal growth within the zone of inhibition measured by the fraction of growth or “FoG”) [8,24]. FoG is quantified relative to the maximum growth achieved by the strain under study on that same plate [24]. As such, this accounts for any differences in growth rates between strains when comparing FoG levels.

**Fig 1:**
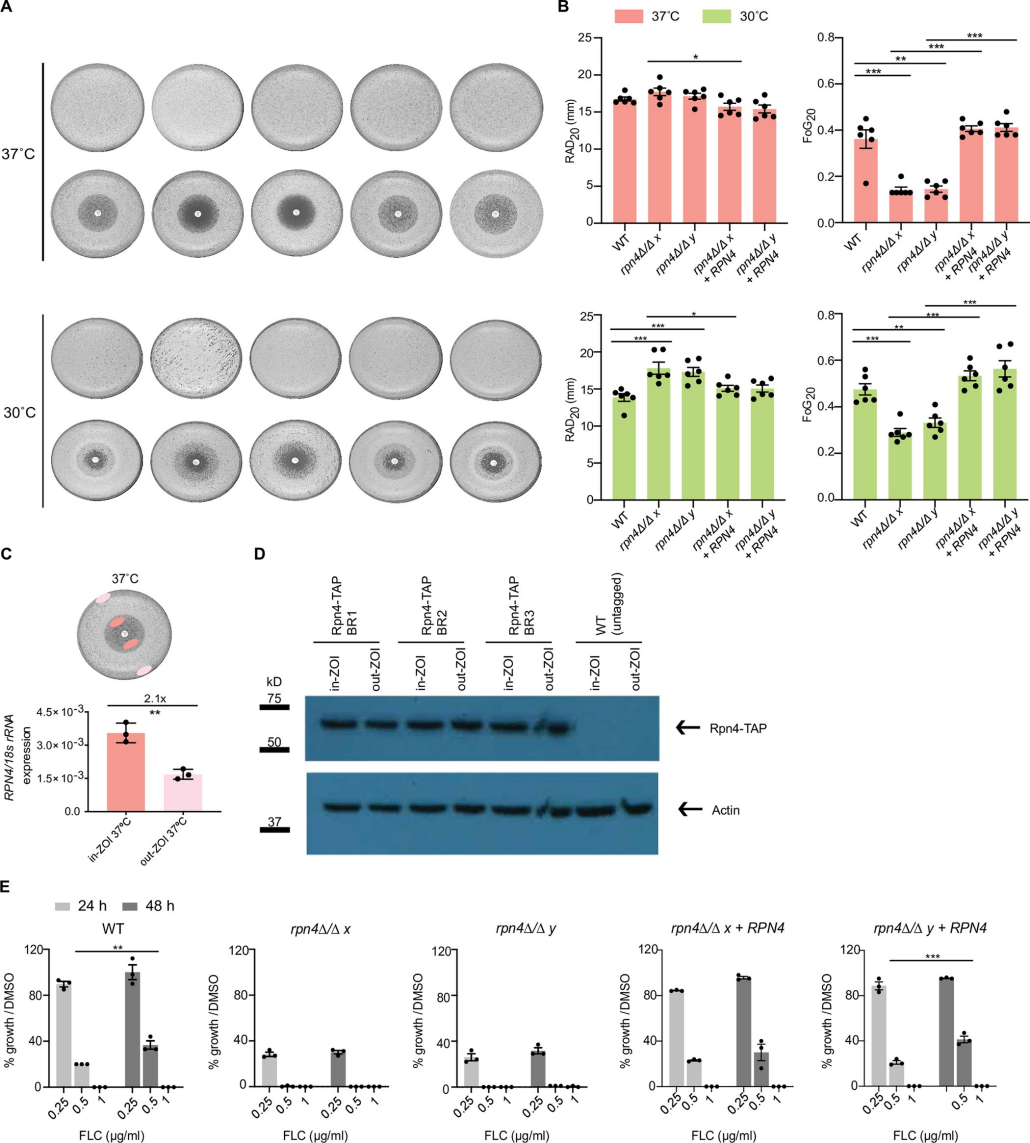
Rpn4 regulates fluconazole susceptibility, with a major role in tolerance. **A.** Fluconazole disk diffusion assays were performed with 25 μg fluconazole disks for wild type (WT), *rpn4Δ/Δ* and complemented strains. Plates were incubated at 30°C or 37°C for 2 days. Six independent experiments were performed and gave equivalent results. One representative experiment is shown. The top panel (without the disk) shows untreated controls (no drug). **B.** *DiskImageR* quantification of RAD_20_ and FoG_20_ for disk diffusion experiments shown in panel A. Data points are from six independent experiments, horizontal bars represent the mean and error bars represent the standard error of mean. * P < 0.05; ** P < 0.01; ***P < 0.001 (2-way ANOVA Bonferroni’s multiple comparison test). Only significant statistical comparisons are shown. **C.** Top: Schematic diagram on the collection of WT ‘tolerant’ cells from within the zone of inhibition and ‘non-tolerant’ cells collected from a fluconazole disk plate grown at 37°C for 2 days. Bottom: qRT-PCR analysis on *RPN4* gene expression was performed from samples collected as described above. Data was normalized to *18S rRNA*. Data points represent three independent experiments, each analysed in two technical replicates. Horizontal bars represent the mean and error bars represent the standard error of mean. ** P < 0.01 (unpaired t-test). **D.** Western blot analysis to detect C-terminally tagged Rpn4 (Rpn4-TAP) was performed on samples collected as described in panel C. Actin served as a loading control. Three independent experiments were performed (BR1 to BR3). The uncropped Western blots are show in S4 Fig. **E.** Percentage growth for WT, *rpn4Δ/Δ* and complemented strains at 0.25, 0.5 and 1 μg/ml fluconazole using the CLSI method, determined relative to the DMSO control. OD_600nm_ was measured after 24 h and 48 h of growth in RPMI media at 35°C. The OD measurements used to determine percentage of growth are given in S3 Dataset. Data points represent three independent experiments, each independent experiment was analysed in two technical replicates. Horizontal bars represent the mean and error bars represent the standard error of mean. ***P < 0.001 (2-way ANOVA Bonferroni’s multiple comparison test). Only significant statistical comparisons are shown.

At 37°C, *rpn4Δ/Δ* showed somewhat increased RAD (Fig 1A and 1B). However, no statistical significance was found for the comparisons of the wild type to the two *rpn4Δ/Δ* deletion clones, although one of the mutant clones (clone x) has a significantly different RAD to its complemented strain (Fig 1A and 1B). In contrast to the relatively minor effect on RAD, there was a significant and large reduction in FoG in both *rpn4Δ/Δ* deletion clones (Fig 1A and 1B). Therefore, the *rpn4Δ/Δ* mutant displays reduced fluconazole tolerance. Complementation with wild type *RPN4* restored FoG for both *rpn4Δ/Δ* deletion clones (Fig 1A and 1B). We also assessed these phenotypes at 30°C and found that the low FoG phenotype of *rpn4Δ/Δ* was less pronounced than at 37°C although the trend was there, and RAD was increased indicating reduced resistance (Fig 1A and 1B). The expression of the *RPN4* gene was elevated in “tolerant” cells growing within the zone of inhibition compared to those growing at the edge of the plate (Fig 1C). However, we could not detect higher Rpn4 protein levels in tolerant cells (Figs 1D and S4), possibly due to Rpn4 being a short-lived protein degraded by the proteasome [25].

We next measured fluconazole susceptibility of *rpn4Δ/Δ* by using the CLSI method, which quantifies growth in liquid RPMI medium at 35°C in the presence of increasing drug concentrations. This analysis showed a two-fold lower fluconazole MIC for *rpn4Δ/Δ* after 24 h of growth (0.5 μg/ml compared to 1 μg/ml for the wild type and complemented strains) (Fig 1E). The MIC after 24 h of growth is a measure of drug resistance [4]. Thus, this result is consistent with a modest role for *rpn4Δ/Δ* in fluconazole resistance as determined by disk diffusion assays shown in Fig 1A and 1B. In the CLSI method, the MIC at 48 h can be used as a measure of drug tolerance [4]. No change in growth was observed for *rpn4Δ/Δ* between 24 and 48 h at the lowest concentration of fluconazole used (0.25 μg/ml, which is approximately the MIC_80_ for the mutant) (Fig 1E). In contrast, the wild type and complemented strains displayed increased growth from 24 to 48 h at their MIC_80_ of 0.5 μg/ml fluconazole (Fig 1E). These data are indicative of tolerant growth occurring in wild type cells, which is absent in *rpn4Δ/Δ*. As such, they support a role for Rpn4 in fluconazole tolerance.

### The transcriptional program regulated by *C*. *albicans* Rpn4

To identify the transcriptional program controlled by Rpn4 in *C*. *albicans*, we used RNA-seq to profile *rpn4Δ/Δ* versus wild type under standard conditions (no stress) and in response to fluconazole. In parallel, we genome sequenced the two *rpn4Δ/Δ* clones. Genome sequencing identified that both clones contain a trisomy of chromosome 7 (S5 Fig). This is in line with common aneuploidies upon generation of mutants in *C*. *albicans*, including in the Homann collection from which our mutants were obtained [26]. The fact that both of the independent *rpn4Δ/Δ* clones maintained an additional chromosome 7 suggest that it might be conferring an advantage. Surprisingly, our analyses of the *rpn4Δ/Δ* RNA-seq data showed no evidence of increased gene expression for chromosome 7 (S6A Fig; mean log fold change -0.05, SEM 0.034), indicating that the additional copy of chromosome 7 is transcriptionally regulated so that gene expression is maintained at normal levels. Although its copy number was not altered in *rpn4Δ/Δ*, there was a shift in gene expression for chromosome 6 (S6A Fig). Further analysis showed that this shift was more prominent in control than fluconazole-treated samples (S6A Fig), and this was due to down-regulation in *rpn4Δ/Δ* in response to fluconazole (S6B Fig). This suggests that Rpn4 controls this set of genes on chromosome 6.

For the RNA-seq, we used a relatively high concentration of fluconazole (3 μg/ml) to mimic drug stress experienced by tolerant cells growing in the zone of inhibition. To reduce the potential for indirect effects caused by long-term fluconazole stress, we limited the time of treatment to 30 minutes. Neither the wild type nor *rpn4Δ/Δ* displayed loss of viability under these treatment conditions (S7 Fig). Three independent experiments were conducted and both independent deletion clones of *rpn4Δ/Δ* were profiled. The transcriptomes of the two mutant clones mostly clustered together and were similar to each other when compared to the wild type, in the presence and absence of fluconazole (S6C and S6D Fig). Therefore, the x and y clone data were considered as replicates and analysed together with an applied batch effect. The entire dataset can be viewed interactively at https://degust.erc.monash.edu/degust/compare.html?code=7626de961da6ddac4911adffc3b6e7ca#/.

**Fig 2:**
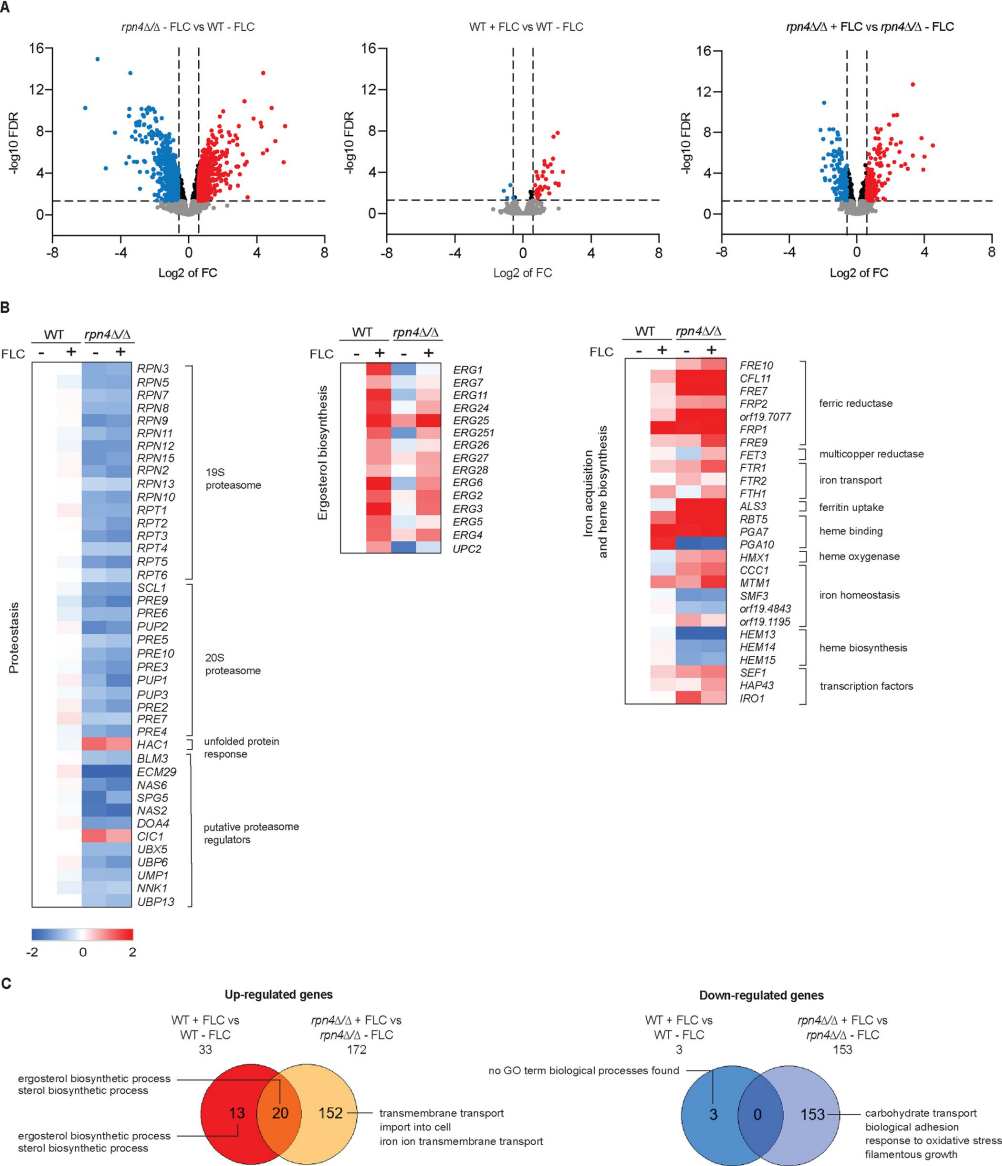
The transcriptional program regulated by *C*. *albicans* Rpn4. **A.** Volcano plot of the RNA-seq data for *rpn4Δ/Δ* relative to wild type (WT) without drug (left graph), WT +/- fluconazole (FLC) (middle graph) and *rpn4Δ/Δ* +/- FLC (right graph); significantly up-regulated genes are highlighted in red, significantly down-regulated genes are in blue. Dotted lines represent the boundary for the fold change cut-off (log2 of 0.585; FDR < 0.05). **B.** Heat map of genes differentially expressed in *rpn4Δ/Δ* cells with functions in proteasome structure and assembly, ergosterol biosynthesis, iron acquisition and heme metabolism. Fold change in gene expression in all samples is expressed relative to the wild type without fluconazole (WT -FLC). Proteosome genes *RPN6*, *RPN1*, *PRE8* and *PRE1* were not included in the heat maps as they fall outside the cut off criteria. The cut-off used is log2 of 0.585, i.e. ≥ 1.5 fold change; FDR < 0.05. **C.** Venn diagram and GO analysis of genes differentially expressed in WT +/- FLC and *rpn4Δ/Δ* +/- FLC (≥ 1.5 fold change; FDR < 0.05). The genes used to construct the Venn diagram and the GO terms for “Process” are detailed in S1 Dataset. The cut-off *p-*value for GO terms was <0.01. The unique genes up-regulated by FLC in *rpn4Δ/Δ* did not show any significant GO terms, except “transmembrane transport” which showed a *p* value of 0.0155. As there are many functionally overlapping GO terms, we did not list all but summarised them in the figure for simplicity. The full GO analysis and list of terms are shown in S1 Dataset.

Under standard growth conditions (without stress), *rpn4Δ/Δ* differentially expressed 1112 genes (FDR 0.05, log2 of 0.585, i.e. 1.5 fold change in gene expression) (Fig 2A, S1 Dataset). Of these, 590 genes were down-regulated and 522 genes were up-regulated. The down-regulated genes showed an enrichment for gene ontology (GO) terms related to the proteasome, proteolysis and ubiquitin-dependent proteolysis (Fig 2B, full list in S1 Dataset). Of the 33 genes encoding proteasome subunits in *C*. *albicans*, 29 were down-regulated in *rpn4Δ/Δ* (Fig 2B, S1 Dataset). In addition, several putative proteasome and ubiquitin-related chaperones and regulators were also down-regulated (Fig 2B, S1 Dataset). The expression of the transcription factor *HAC1* (which regulates the unfolded protein response) was induced (Fig 2B).

Further to the proteasome genes, *rpn4Δ/Δ* cells displayed lower transcript levels for several ergosterol biosynthesis genes and their transcriptional activator *UPC2* (Fig 2B, S1 Dataset). The GO term “sterol metabolic process” was enriched with a *p* value of 0.04995 (S1 Dataset). The enzymes of heme biosynthesis *HEM13*, *HEM14* and *HEM15* were also down-regulated (Fig 2B, S1 Dataset) (note that heme is a co-factor for ergosterol biosynthesis) [27]. In contrast, several genes needed for iron uptake, including the iron-regulated transcription factors *SEF1*, *HAP43* and *IRO1*, were up-regulated (Fig 2B). We note however that some of these iron-related genes were expressed at low levels (e.g. *CFL11*) and the only GO term related to metals that was significantly enriched in the *rpn4Δ/Δ* down-regulated gene set was “metal iron binding” (*p* = 0.01433, S1 Dataset). In addition to these pathways, GO categories related to carbon metabolism (including “glycolytic process”, “ATP generation from ADP” and “pyruvate metabolic process”), autophagy, vacuolar transport, organonitrogen compound metabolic process and the ESCRT complex were also enriched in the genes down-regulated in *rpn4Δ/Δ* (S1 Dataset).

Functional groups up-regulated in *rpn4Δ/Δ* cells include GO terms related to ribosome biogenesis, rRNA and ncRNA processes (S1 Dataset). Although there was no significant enrichment in GO terms related to hyphae, several cell surface proteins and hyphal-specific genes were up-regulated (S1 Dataset). This includes the genes determined by Carlisle and Kadosh to represent the core hyphae-induced transcripts [28], such as *ALS3*, *HWP1*, *ECE1*, *HYR1*, *RBT5*, *IHD1* and the activator *UME6* (S1 Dataset). Up-regulation of hyphae-specific genes likely reflects the hyper-filamentous morphology of *rpn4Δ/Δ* cells (S2 Fig).

We next performed ChIPseq in order to determine the direct targets of *C*. *albicans* Rpn4. Unfortunately, these attempts failed with two different C-terminal tags on Rpn4 (TAP and HA), and in both standard growth conditions (without drug stress) and following the addition of fluconazole. A possible explanation for ChIPseq failure is a transient interaction of Rpn4 with DNA. We propose this because in *S*. *cerevisiae* Rpn4 has a short half-life due to a regulatory feedback loop whereby it is degraded by the proteasome [25]. It is reasonable to assume that similar regulation could be occurring in *C*. *albicans*. As the next best thing, we performed a bioinformatic search for Rpn4 binding sites in the upstream regulatory regions of *C*. *albicans* genes (+1kb). For this, we used the *C*. *albicans* Rpn4 binding site determined by Gasch et al [29]: AGTGGCAAAN, GGTGGCAAYA, GRAGGCAAAA. This search identified 217 putative Rpn4 targets (S2 Dataset). Of these, 64 were differentially expressed in *rpn4Δ/Δ* (56 under basal conditions, and 8 after addition of FLC). The majority of these putative Rpn4 targets (46 out of 64) were down-regulated in the mutant (S2 Dataset), suggesting that *C*. *albicans* Rpn4 acts predominantly as a transcriptional activator. Of the proteasome genes, 18 had a Rpn4 binding site. Another 10 were identified when we relaxed the search to include less common binding sites according to the matrix reported in Gasch et al (RRWGGCAAHN) [29]. Of the *ERG* and *HEM* genes only *ERG7* and *HEM15* had a Rpn4 binding site, but in both cases over 950 bp upstream of the start codon (S2 Dataset).

### Rpn4 contributes to fluconazole susceptibility by controlling ergosterol gene expression

Next we turned our efforts to the fluconazole response. Fluconazole-treated wild type cells differentially expressed 36 genes and, as expected, activated the ergosterol biosynthesis pathway (Fig 2A (middle panel), 2B and S1 Dataset). No other major changes were observed, likely due to our short treatment time of 30 minutes. The transcriptional response of *rpn4Δ/Δ* upon fluconazole treatment was more extensive, with 325 genes differentially expressed when comparing treated versus untreated mutant (Fig 2A right panel and S1 Dataset). Of the 172 genes up-regulated in *rpn4Δ/Δ*, 20 were shared with the wild type (Fig 2C). Ergosterol biosynthesis genes were enriched in this shared group (Fig 2C). Genes up-regulated by fluconazole in *rpn4Δ/Δ* also displayed an enrichment for categories related to import, including “iron import into cell” (S1 Dataset). However iron import genes *FTH1*, *FTR1*, *SIT1*, *FRP1* and *FET3* were mostly expressed at low levels and/or modestly up-regulated in the *rpn4Δ/Δ* mutant (see data at https://degust.erc.monash.edu/degust/compare.html?code=7626de961da6ddac4911adffc3b6e7ca#/).

There were only 3 genes whose expression was reduced by fluconazole in the wild type, while in *rpn4Δ/Δ* there were 153 genes (Fig 2C). Of these, hyphal genes were down-regulated (S1 Dataset). This is consistent with the fact that fluconazole represses hyphal morphogenesis [30]. Several other GO terms also showed reduced expression in fluconazole-treated *rpn4Δ/Δ* cells, including carbohydrate transport, biofilm formation and aggregation (Fig 2C). There were no changes to the expression of heme biosynthesis genes in response to fluconazole, in either the wild type or *rpn4Δ/Δ* strains (Fig 2B, S1 Dataset). Similarly, the proteasome genes remained expressed at low levels in *rpn4Δ/Δ*, in the presence or absence of fluconazole (Fig 2B).

**Fig 3:**
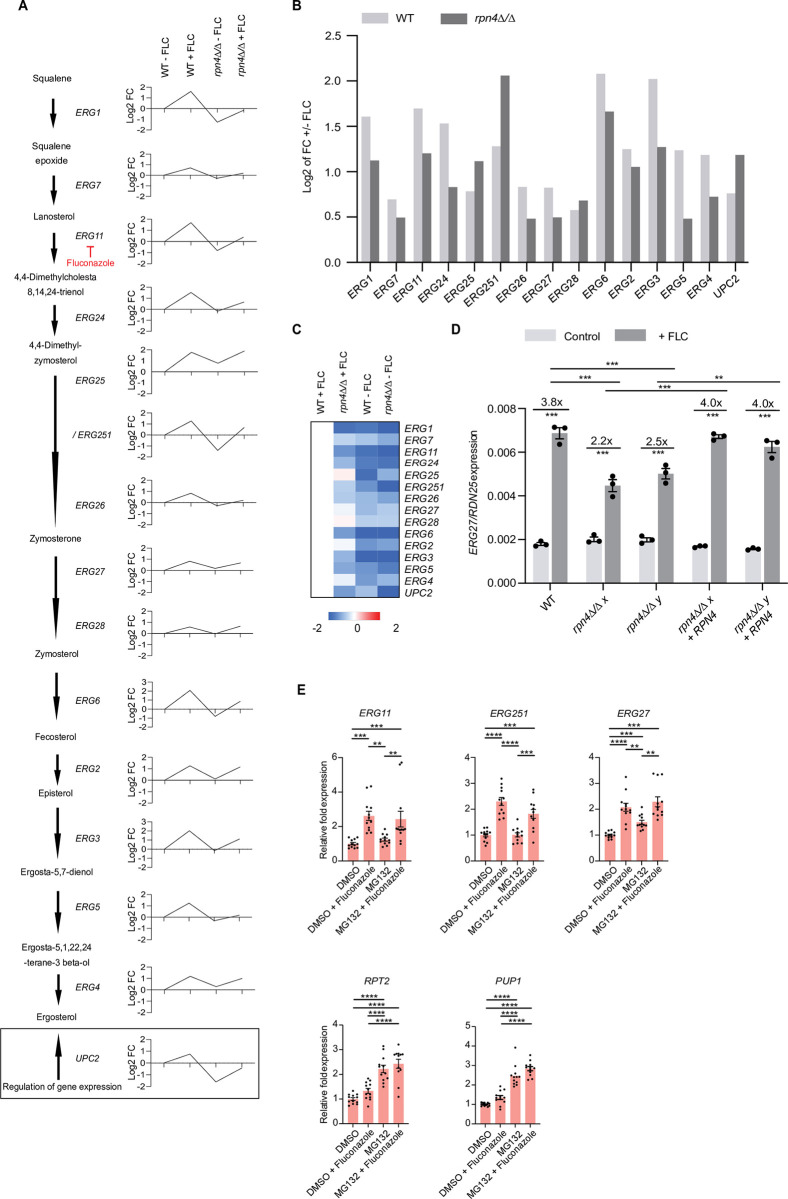
The roles of Rpn4 in ergosterol gene expression. **A.** Expression of the *ERG* genes and *UPC2* in the RNA-seq experiment in wild type (WT) and *rpn4Δ/Δ* in the absence and presence of fluconazole (FLC). Shown are the log2 values of fold change relative to untreated (-FLC) WT, which is set to 1 (log2 of 0). The ergosterol biosynthesis pathway is shown on the left for context. **B.** Comparison of fold change (expressed as log2 values) in the expression of the *ERG* genes and *UPC2* in wild type and *rpn4Δ/Δ* in response to FLC. **C.** Same heat map as the ergosterol biosynthesis genes shown in Fig 2B but here shown as fold change in gene expression relative to wild type +FLC. Cut-offs were 1.5 fold change; FDR < 0.05. **D.** qPCR analysis of *ERG27* gene expression in WT, *rpn4Δ/Δ* and complemented strains. Gene expression was normalized to *RDN25*. Data points represent three independent experiments, each analysed in two technical replicates. Horizontal bars represent the mean and error bars represent the standard error of mean. ** P < 0.01; *** P < 0.001 (2-way ANOVA Bonferroni’s multiple comparison test). Only significant statistical comparisons are shown. **E.** qPCRs of proteasome and *ERG* gene expression in response to proteasome inhibition with MG132. Wild type *C*. *albicans* was treated with 50 μM MG132 for 3 h, followed by 30 min of fluconazole (FLC) treatment (3 μg/ml). Gene expression was normalized to *RDN25* and expressed relative to the average expression in the untreated wild type (See S3 Dataset). Data points represent biological repeats from 4 independent experiments. In each experiment, 3 independent colonies were cultured for each condition, leading to 12 repeats. The horizontal bars represent the mean and error bars represent the standard error of mean. ** P < 0.01; *** P < 0.0001 (2-way ANOVA Bonferroni’s multiple comparison test). Only significant statistical comparisons are shown.

We next studied the *ERG* genes more closely, since some of them were down-regulated in *rpn4Δ/Δ* under basal levels but up-regulated by fluconazole (Fig 3A). This analysis showed that *rpn4Δ/Δ* could upregulate the *ERG* genes in response to fluconazole but their up-regulation in many cases was smaller than in the wild type (exceptions to this were *ERG25*, *ERG28* and *UPC2*) (Fig 3B). In other words, regardless of their upregulation, many of the *ERG* genes and their transcription factor *UPC2* remained expressed at lower levels in fluconazole-treated *rpn4Δ/Δ* versus fluconazole-treated wild type (Fig 3C). qRT-PCR experiments confirmed activation of *ERG27* in the wild type by fluconazole and reduced activation in *rpn4Δ/Δ* (Fig 3D). The complemented strains restored *ERG27* transcriptional activation to wild type levels (Fig 3D).

The proteasome is a key transcriptional target of *C*. *albicans* Rpn4 (Fig 2B). Given the lack of Rpn4 binding sites upstream of the *ERG* genes in our bioinformatic analysis (S2 Dataset), we were wondering if the regulation of *ERG* gene expression by Rpn4 is controlled indirectly, via its roles in proteasome function. To test this, we treated wild type cells with the proteasome inhibitor MG132, and then profiled *ERG* gene expression with and without fluconazole. As a control for MG132 activity, we show that MG132 caused an upregulation of proteasome genes *RPT2* and *PUP1*, likely as a compensatory response to proteasome inhibition (Fig 3E). At basal levels (without fluconazole), the expression of *ERG11*, *ERG251* and *ERG27* did no change in response to proteasome inhibition with MG132 (Fig 3E, -FLC samples). This is in contrast to the down-regulation of *ERG251* and *ERG11* in *rpn4Δ/Δ* (Fig 3A). In response to fluconazole, MG132-treated cells were able to upregulate the *ERG* genes (Fig 3E). We noticed that there some variability in ERG gene activation in MG132 and FLC-treated cells likely due to heterogenous stress responses. We therefore performed four independent experiments, with 3 independent cultures per group in each (i.e. 12 biological repeats). While in some experiments, MG132-treated cells showed reduced *ERG* gene activation in response to fluconazole (see S3 Dataset), when taken together there were no major differences (Fig 3E). These data suggest that Rpn4 has proteasome-independent roles in *ERG* gene expression.

**Fig 4:**
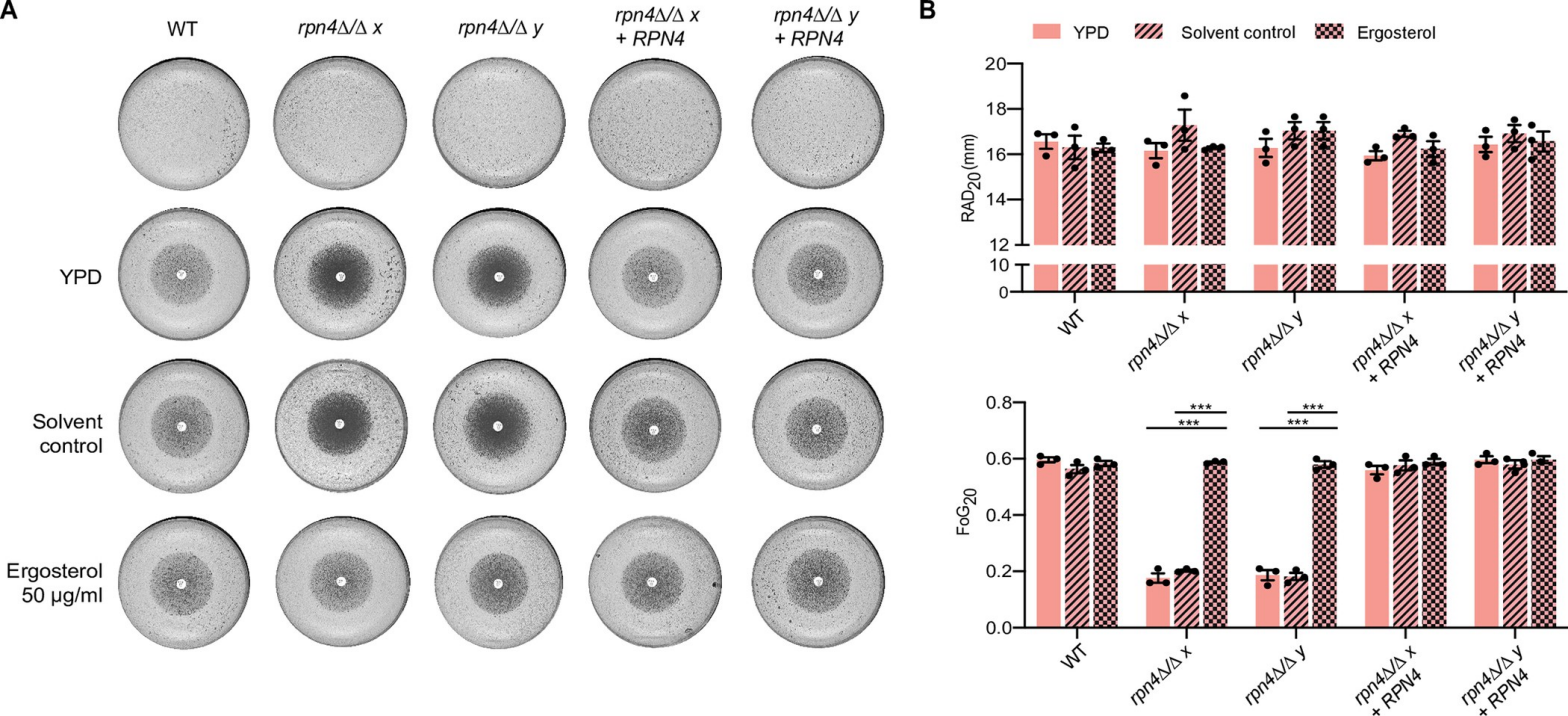
Rpn4 contributes to fluconazole tolerance *via* ergosterol supply. **A.** Fluconazole disk diffusion assays (25 μg fluconazole/disk) for wild type (WT), *rpn4Δ/Δ* and complemented strains. Some plates were supplemented with 50 μg/ml ergosterol or an equivalent amount of the drug solvent (1:1 Tween80/Ethanol, final concentration 1.25%). Three independent experiments were performed and gave equivalent results. One representative experiment is shown. The top panel (without the disk) shows untreated conditions (no drug). **B.** *DiskImageR* analysis of RAD_20_ and FoG_20_ values for the experiments described in panel A. Data points represent three independent experiments, horizontal bars represent the mean and error bars represent the standard error of mean. *** P < 0.001 (2-way ANOVA Bonferroni’s multiple comparison test). Only significant statistical comparisons are shown.

To address the functional relevance of lower *ERG* gene expression in *rpn4Δ/Δ* we made use of the fact that *C*. *albicans* is capable of sterol uptake from the medium [31] and asked if supplementing ergosterol could restore fluconazole tolerance in the mutant. Indeed, this was the case: ergosterol restored growth of *rpn4Δ/Δ* in the zone of inhibition and FoG_20_ levels of *rpn4Δ/Δ* in the presence of ergosterol were the same as for the wild type (Fig 4A and 4B). Collectively, our findings indicate that one of the mechanisms by which Rpn4 regulates fluconazole tolerance is by enabling ergosterol supply *via ERG* gene expression.

### Functions of the proteasome in fluconazole tolerance and resistance

**Fig 5:**
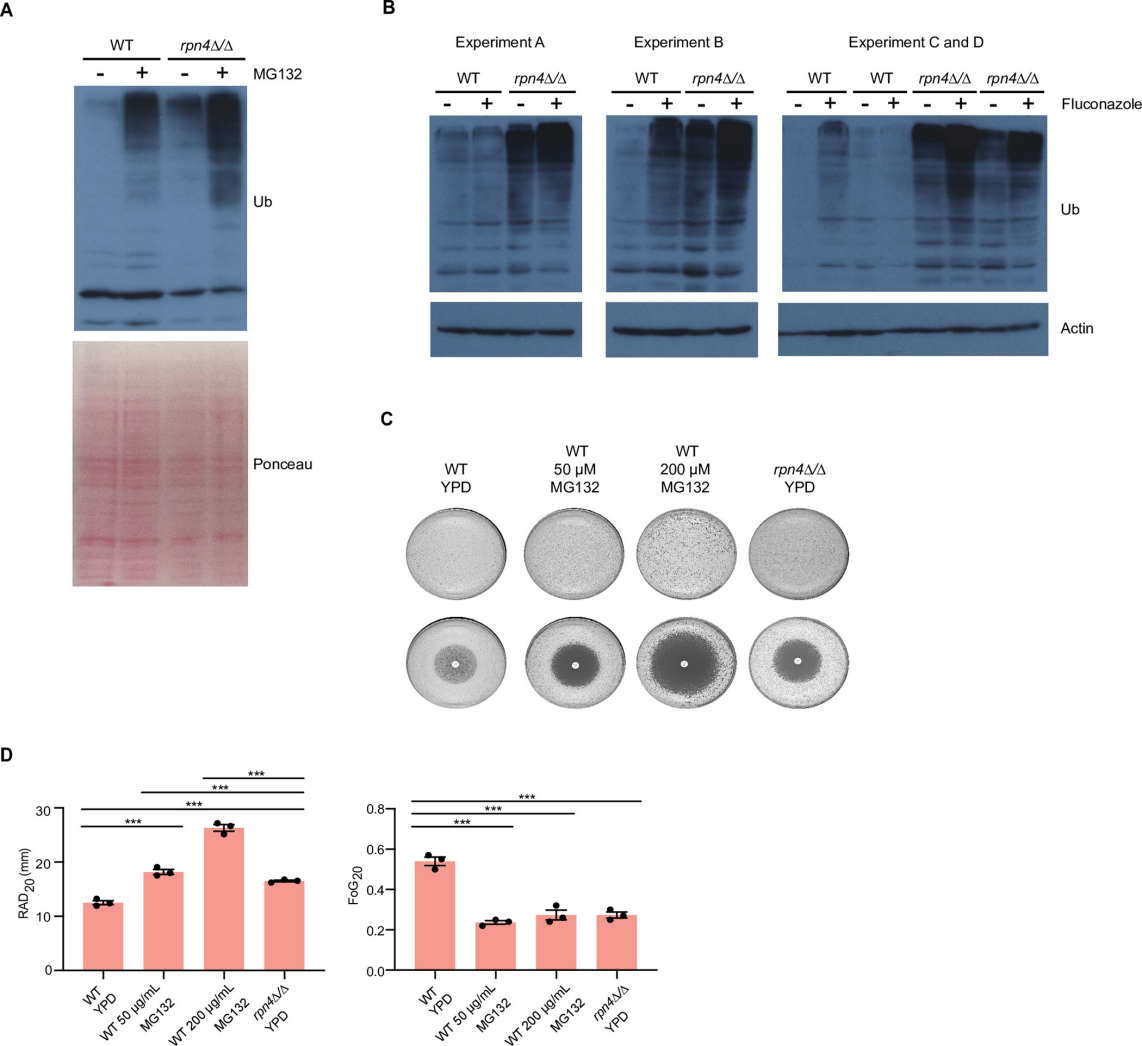
Roles of the proteasome in fluconazole tolerance and resistance. **A.** Western blot using the anti-Ubiquitin antibody in extracts from wild type (WT) and *rpn4Δ/Δ* cells, with or without MG132 (50 μM). Ponceau stained membrane is shown as the loading control. **B.** Western blot using the anti-Ubiquitin antibody in extracts from wild type (WT) and *rpn4Δ/Δ* cells, with or without fluconazole (FLC) treatment. Actin is shown as the loading control. Four biological repeats were performed and are shown here. Repeats C and D were done together in the same experiment, with independent cultures for each of the strains. **C.** Fluconazole disk diffusion assays (25 μg fluconazole/disk) in the presence or absence of MG132 at the indicated concentrations. The *rpn4Δ/Δ* strain is shown as control. Plates were incubated at 30°C or 37°C for 2 days. Three independent experiments were performed and gave equivalent results. One representative experiment is shown. The top panel (without the disk) shows untreated conditions (no drug). **D.** *DiskImageR* analysis for RAD_20_ and FoG_20_ values of the MG132 experiments shown in panel C. Data points represent three independent experiments, horizontal bars represent the mean and error bars represent the standard error of mean. *** P < 0.001 (2-way ANOVA Bonferroni’s multiple comparison test). Only significant statistical comparisons are shown.

As shown by our RNAseq data, the proteasome is a key transcriptional target of *C*. *albicans* Rpn4 (Fig 2B). Proteasome genes are down-regulated in *rpn4Δ/Δ* cells (Fig 2B), and there is an accumulation of ubiquitinated proteins, consistent with lower proteasome activity in the mutant (Fig 5A). The levels of ubiquitinated proteins in *rpn4Δ/Δ* cells were higher than in wild type cells, but lower than in wild type cells treated with the proteasome inhibitor MG132 (Fig 5A). Applying MG132 to *rpn4Δ/Δ* cells resulted in a further accumulation of ubiquitinated proteins (Fig 5A). Collectively, these data show that proteasome activity is diminished in the *C*. *albicans rpn4Δ/Δ* mutant, but it is not completely inactivated.

Next we asked if fluconazole causes proteasome stress by perturbing protein homeostasis. We did this by studying the accumulation of ubiquitinated proteins in response to fluconazole. In wild type cells, there was a heterogeneous response. In two out of four biological repeats fluconazole induced a clear accumulation of ubiquitinated proteins (Fig 5B, Experiment B and C). In the other two repeats (Experiment A and D), we did not observe a convincing accumulation (Fig 5B), suggesting that cells adapted and dealt effectively with the missfolded proteins. In contrast to the wild type, in *rpn4Δ/Δ* cells there was a reproducible accumulation of ubiquitinated proteins in response to fluconazole above what is seen in the mutant without stress (Fig 5B). Consistent with an important role for the proteasome in fluconazole responses, MG132 impaired both resistance and tolerance to fluconazole, as shown by increased RAD and reduced FoG in fluconazole disk diffusion assays (Fig 5C and 5D). The reduction in FoG by MG132 phenocopied *rpn4Δ/Δ* (Fig 5C and 5D). Interestingly, unlike *rpn4Δ/Δ*, the ubiquitin mutant *ubi4Δ/Δ* showed no changes to fluconazole FoG at 37°C, although RAD was increased (S8A and S8B Fig). The lack of fluconazole FoG phenotype of *ubi4Δ/Δ* is possibly due to compensation from *UBI3* [32]. Unfortunately, we could not test this hypothesis as *UBI3* is an essentially gene and therefore a double *ubi4Δ/Δ ubi3Δ/Δ* mutant could not be constructed. Collectively, the results presented in Fig 5 indicate that Rpn4-dependent regulation of the proteasome is critical for providing enough capacity for cells to deal with accumulation of misfolded proteins and proteotoxicity caused by fluconazole. The ability to maintain protein homeostasis is essential for both tolerance and resistance to fluconazole.

## Discussion

Here we identify the transcriptional activator Rpn4 as a regulator of fluconazole tolerance in *C*. *albicans*. Our analysis of a range of *rpn4Δ/Δ* mutant phenotypes showed that the strongest defect was its hyper-susceptibility to fluconazole. Since *C*. *albicans* overcomes fluconazole by a complex set of mechanisms involving both drug resistance and drug tolerance [4], we utilised assays that distinguish between these two facets of drug susceptibility. Using these assays, we found that Rpn4 is essential for fluconazole tolerance. This was especially true at the clinically relevant temperature of 37°C, at which tolerant growth was practically non-existent in the mutant. In contrast, Rpn4 plays a more minor role in fluconazole resistance, with only a two-fold change in the fluconazole MIC for *rpn4Δ/Δ*. We acknowledge that *rpn4Δ/Δ* grows more slowly than the wild type even without stress (15% reduced growth rate) (S1B and S1C Fig). However, we argue that a generalised growth defect does not explain the large drop in fluconazole tolerance observed for *rpn4Δ/Δ*. First, the reduction in tolerant growth of the mutant is dramatic, while the growth defect is minor. Second, tolerant growth is measured as a fraction of growth relative to maximal growth achieved on the same plate, thereby accounting for differences in growth rates between fungal strains [24]. Instead, we propose that Rpn4-activated pathways mitigate fluconazole stress in two ways: Rpn4 activates proteasome gene expression for overcoming drug-induced proteotoxicity, and it further contributes to the expression of ergosterol genes to mitigate ergosterol biosynthesis inhibition and membrane stress caused by fluconazole.

With respect to ergosterol biosynthesis, our transcriptome data showed that Rpn4 promotes constitutive transcription of ergosterol and heme biosynthesis genes, and is also required for full induction of several ergosterol genes by fluconazole. A role for Rpn4 in alleviating fluconazole-induced ergosterol depletion is shown by the fact that supplementation of ergosterol to *rpn4Δ/Δ* restores fluconazole tolerance (Fig 4). Our data is supported by a recent study showing that Rpn4 activates ergosterol and heme gene expression in another yeast pathogen, *Candida glabrata* [33]. However, the regulatory mechanisms are somewhat different. In contrast to what we show in *C*. *albicans*, *C*. *glabrata* Rpn4 is not required for constitutive expression of the ergosterol or heme genes, only for their induction by fluconazole [33]. Also, *C*. *glabrata* Rpn4 binds directly to the *ERG11* promoter, which contains an Rpn4-recognition motif [33], but we found no Rpn4 binding sites in *ERG11* or other *ERG* genes suggesting that in *C*. *albicans* their regulation by Rpn4 is indirect. We considered that lower proteasome activity might be the reason for reduced *ERG* gene expression in *rpn4Δ/Δ* cells. Our data argues against that possibility, as expression we detected normal of *ERG* transcript levels upon proteasome inhibition with MG132 (Fig 3D). The expression of *UPC2*, encoding the central transcriptional activator of ergosterol biosynthesis in *C*. *albicans*, was down-regulated in *rpn4Δ/Δ* cells (Fig 2B). This suggests that Rpn4 might be regulating the *ERG* genes by activating *UPC2*. However, we found no Rpn4 binding sites in the *UPC2* promoter. It remains to be determined how *C*. *albicans* Rpn4 regulates the expression of *ERG* and *HEM* genes.

Regulation of proteasome gene expression is the best-known function of Rpn4 in yeasts [15,25]. A role for *C*. *albicans* Rpn4 in proteasome regulation has been suggested by bioinformatic analyses, which found Rpn4 binding sites upstream of proteasome genes [29]. Our data now provides experimental evidence that *C*. *albicans* Rpn4 activates proteasome gene expression under standard growth conditions and under drug stress, and we also show that proteasome activity is reduced in *rpn4Δ/Δ* cells. The reduction in proteasome activity sensitizes *rpn4Δ/Δ* cells to fluconazole-induced proteotoxicity, as shown by an accumulation of ubiquitinated proteins in fluconazole-treated *rpn4Δ/Δ* cells. Moreover, the reduction in fluconazole tolerance of *rpn4Δ/Δ* was more pronounced at 37 than at 30°C (Fig 1). This is also consistent with a key role of proteostasis in fluconazole tolerance, as the higher temperature of 37°C is expected to exacerbate proteotoxicity from unfolded protein accumulation, making the loss of proteasome activity in *rpn4Δ/Δ* more evident. Collectively, our data demonstrates that Rpn4-dependent regulation of the proteasome is critical for providing sufficient proteasome capacity to overcome fluconazole-induced proteotoxicity. Supporting this hypothesis, proteasome inhibition by MG132 had a large effect on fluconazole tolerance as well as resistance, and phenocopied *rpn4Δ/Δ* for reduced tolerance (Fig 5). Rpn4 plays a role in fluconazole susceptibility in two other yeasts, *C*. *glabrata* [33] and *S*. *cerevisiae* [34,35]. As such, we propose that the roles of Rpn4-regulated proteasome functions in azole responses are conserved across yeast species.

How does Rpn4 control fluconazole tolerance *via* the proteasome? There are several possible scenarios. Low proteasome activity and unfolded protein accumulation in *rpn4Δ/Δ* could tie up the Hsp90 chaperone, preventing it from exerting its functions in fluconazole tolerance [11–13,36]. However, *rpn4Δ/Δ* cells were not hypersusceptible to the Hsp90 inhibitor geldanamycin (S1A Fig), suggesting that Rpn4 has Hsp90-independent functions. A second possibility is suggested by data from *S*. *cerevisiae*, which showed that the proteasome degrades ergosterol biosynthesis enzymes [37–39]. This process ensures sterol homeostasis by limiting the activity of the ergosterol biosynthesis pathway to avoid the accumulation of toxic sterol intermediates [38,39]. It is conceivable (and likely) that similar mechanisms operate in *C*. *albicans*. Therefore, we speculate that Rpn4-dependent regulation of the proteasome might control Erg enzyme levels to reduce toxic sterols, which would be particularly important in response to blockade of the ergosterol biosynthesis pathway by fluconazole. Supporting this hypothesis, it has been shown that the *S*. *cerevisiae rpn4* mutant displays higher levels of several ergosterol biosynthesis enzymes due to their reduced protein degradation [40].

How does this hypothesis fit with our data showing lower *ERG* gene transcripts in the *C*. *albicans rpn4Δ/Δ* mutant? We considered the possibility of feedback regulation: if the *C*. *albicans rpn4Δ/Δ* mutant displays higher Erg protein levels due to lower proteasomal degradation (similar to what has been shown in *S*. *cerevisiae*), then cells could try to compensate by reducing *ERG* gene transcription to restore sterol homeostasis. While we cannot exclude this possibility, our data argues against it because we show that treatment of wild type *C*. *albicans* with the proteasome inhibitor MG132 does not reduce *ERG* transcript levels (Fig 3). Thus, we propose that the role of Rpn4 in proteasome regulation is distinct from its role in *ERG* gene expression. That said, it would make sense for *C*. *albicans* to use Rpn4 to coordinate *ERG* gene expression with proteasome-dependent degradation of the Erg enzymes. This sort of regulation would represent an important homeostatic mechanism to ensure optimal activity of the ergosterol biosynthesis pathway, so that toxic sterol intermediates are kept in check but ergosterol is supplied to membranes to enable cell growth. Indeed, our data shows that the homeostatic mechanisms regulated by *C*. *albicans* Rpn4 are essential for overcoming growth inhibition by fluconazole, which is important for drug tolerance.

The proteasome is a therapeutic target in human diseases, and Rpn4 is a fungal-specific transcriptional activator with no direct homologs in mammals. Therefore, our results shed new light on the mechanisms of antifungal drug tolerance in *C*. *albicans* and identify possible targets for therapeutic intervention.

## Materials and methods

### Ethics statement

All animal experiments were approved by the Monash University Animal Ethics Committee (approval numbers ERM14292 and ERM25488).

### *C*. *albicans* strains

The strains used in this study are shown in S1 Table. Two independent deletion clones of *RPN4*, *rpn4Δ/Δ* x and y (*HIS1*^*+*^ *LEU2*^*+*^ *arg4*^*-*^) were obtained from a transcriptional regulator knockout library [22], which was obtained from the Fungal Genetics Stock Centre [41]. These *rpn4Δ/Δ* mutant clones are homozygous deletion strains in which *RPN4* was deleted by utilizing the *C*. *dubliniensis HIS1* and *C*. *maltose LEU2* markers [42]. The deletion clones were genotyped by PCR to confirm homozygous deletion of *RPN4* and were also reversed to arginine prototrophy by insertion of *C*. *dubliniensis ARG4* into the *LEU2* locus to become *HIS1*^*+*^ *LEU2*^*+*^ *ARG4*^*+*^. To construct the *RPN4*-complement strain, one copy of wild type *RPN4* was introduced at the *LEU2* locus of *rpn4Δ/Δ* using the *C*. *dubliniensis ARG4* marker. In addition, a second wild type *RPN4* copy was introduced at the endogenous *RPN4* locus upstream of the *HIS1* disruption cassette using the *NAT1* selectable marker. Genome sequencing of the strains identified that both deletion clones (x and y) contained a trisomy of chromosome 7, which was however transcriptionally buffered (S5 and S6 Figs). For all of the above strains, the wild type reference SN425 (*HIS1*^*+*^ *LEU2*^*+*^ *ARG4*^*+*^), a phototrophic strain derived from SN152, was used.

The *ubi4Δ/Δ* mutant (*HIS1*^*+*^ *LEU2*^*+*^ *arg4*^*-*^) was generated in this study from strain SN152 (*his1*^*-*^ *leu2*^*-*^ *arg4*^*-*^). Homozygous deletion of *ubi4Δ/Δ* was produced by deletion of *UBI4* utilizing the *C*. *dubliniensis HIS1* and *C*. *maltose LEU2* markers. For the *ubi4Δ/Δ* strain, the wild type reference SN250 (*HIS1*^*+*^ *LEU2*^*+*^ *arg4*^*-*^), a strain derived from SN152, was used. Two independent knockout clones of *ubi4Δ/Δ* were analysed in the experiments. Rpn4-TAP (*HIS1*^*+*^ *leu2*^*-*^ *ARG4*^*+*^*)* was constructed by introducing a TAP tag amplified from plasmids pFA-TAP-*HIS1* or pFA-TAP-*ARG4* by PCR to the C-terminus of *RPN4* in the parental strain SN152 [43]. Both alleles were tagged. For the Rpn4-TAP strain, the wild type reference SN425 (*HIS1*^*+*^ *LEU2*^*+*^ *ARG4*^*+*^) was used. All transformants were confirmed by diagnostic PCR (for primers see S2 Table).

### Growth conditions for fungal cultures

For standard growth, *C*. *albicans* strains were grown in YPD medium (1% yeast extract, 2% peptone, 2% glucose, 80 μg/ml uridine, with addition of 2% agar for plates) at 30°C with shaking at 200 rpm. For yeast growth, overnight-cultured strains were diluted to OD_600nm_ of 0.2 into prewarmed YPD medium at 30°C with shaking at 200 rpm. For hyphal growth, overnight-cultured strains were diluted to OD_600nm_ of 0.2 into prewarmed bone-marrow-derived mouse macrophages (BMDM) medium containing serum-free RPMI 1640 media pH 7.4 supplemented with 10 mM glucose at 37°C with shaking at 200 rpm. Ergosterol (Sigma-Aldrich) was supplemented onto YPD plates to determine the effect of exogenous ergosterol on fluconazole susceptibility. A solvent control of 1:1 Tween80/Ethanol was included. For determining cell viability following fluconazole treatment, overnight-cultured strains were diluted to OD_600nm_ of 0.2 and grown to log phase (OD_600nm_ of 1) in YPD medium at 37°C with shaking at 200 rpm. Cells were then treated with 3 μg/ml fluconazole or the solvent control DMSO (Sigma-Aldrich, final concentration 0.06%) for 30 min. Cultures were serially diluted and plated on YPD agar plates. Colonies were counted after 2 days of incubation at 30°C.

### Macrophage infection experiments and live cell microscopy

Bone marrow were extracted from femur and tibia bones of male or female 6 to 8 weeks old C57BL/6 mice from the Monash Animal Research Platform. Extracted monocytes were suspended in differentiation medium: RPMI 1640 pH 7.4 supplemented with supplemented with 10mM glucose, 12.5mM HEPES, 15% fetal bovine serum (Serana), 20% L-cell conditioned medium containing macrophage colony-stimulating factor and 100 U/mL of penicillin-streptomycin (Sigma-Aldrich), and differentiated into mouse bone marrow-derived macrophages (BMDMs) for 5 to 7 days, as previously described [44]. A cell scraper was used to scrape differentiated macrophages from petri dishes. Macrophages were then seeded onto 96-well microplates at a density of 5 x 10^5^ cells/well and incubated overnight at 37°C in 5% CO_2_. Before fungal challenge, macrophages were stained with 1 μM CellTracker Green CMFDA dye (Thermo Fisher Scientific) for 20 min in serum-free RPMI medium 1640 pH 7.4 lacking glucose. To prepare *C*. *albicans* for macrophage infections, single colonies were patched onto YPD plates and incubated at 30°C overnight. *C*. *albicans* cells were scrapped off plates, resuspended in PBS and inoculated at 7.5 x 10^4^ cells/ml in differentiation medium (described above). Macrophages were then infected by replacing serum-free CellTracker Green medium with the differentiation medium (as described above) containing *C*. *albicans* cells at a multiplicities of infection (MOI) of 1.5:1 for S3B Fig. Phagocytosis was allowed to proceed for 1 h, followed by washing of non-phagocytosed fungal cells with PBS. Mouse BMDM differentiation medium was then added to each well, supplemented with 10 mM glucose. To track dead macrophages, 0.6 μM DRAQ7 (Abcam) was added to the medium. Live cell imaging was performed at 37°C in 5% CO_2_ as previously described [21,44]. A minimum of 1000 macrophages were counted for each biological replicate. Imaging was performed using a Leica DMi8 with a GTC9000 camera and Leica LAS X software. Fluorescence excitation needed to image cell death nuclei (DRAQ7) was achieved using the Leica EL6000 external light source with a Y5 filter cube. All images were captured using the HC PL FLUOTAR L 20x / 0.40 Dry PH1 CORR objective. The data were analysed and quantified using CellProfiler (version 2.1.1) and FIJI image analysis programme [45]. Selected microscopic positions were excluded based on counting irregularities caused by out-of-focus images. Snapshots of the live cell imaging data were obtained using FIJI.

To calculate the phagocytic index (S3A Fig), macrophages were infected with *C*. *albicans* cells at a MOI of 2:1. At 1 h post-phagocytosis, macrophages were washed with PBS to remove un-phagocytosed *C*. *albicans* and fixed with 4% paraformaldehyde. Following fixation, macrophages were permeabilised using 0.1% Triton-X 100 and then stained with 10 μg/ml calcofluor white to count the phagocytosed *C*. *albicans* cells. A minimum of 1800 macrophages were counted for each biological replicate.

For determining glucose concentrations in the macrophage infection medium (S3C Fig), infections were performed as above (MOI 1.5 of wild type or *rpn4Δ/Δ C*. *albicans*:1 macrophage) and samples taken after 1, 12 and 14 h. The concentration of glucose was determined using the glucose oxidase kit (Amplex Red, Thermofisher), following the manufacturer’s instructions.

### Quantitative PCR analysis of gene expression

Overnight-grown cultures of *C*. *albicans* strains were diluted to an OD_600nm_ of 0.2 and grown in 10 or 20 ml of YPD medium at 37°C with shaking at 200 rpm. Total RNA was extracted using the hot phenol method as we described previously [46]. One-μg of DNase I-treated total RNA was reverse-transcribed using SuperScript III Reverse Transcriptase (Invitrogen) according to the manufacturer’s instructions. qRT-PCR reactions were performed on the LightCycler 480. Data were analysed with the LinReg software (version 11.0) [47]. At least three biological repeats were performed per experiment with two technical repeats (number of repeats is indicated in the figure legends). Statistical analysis on biological repeats was performed on the normalized expression values to either the *RDN25* or the *18s rRNA* gene. For detecting the expression levels of *RPN4* in Fig 1C, ‘tolerant’ wild type cells from within the zone of inhibition and ‘non-tolerant’ cells outside the zone of inhibition were collected from a fluconazole disk plates grown at 37°C for 2 days and resuspended in PBS. For detecting the expression levels of *ERG27* in Fig 3D, overnight-grown cultures of *C*. *albicans* strains were diluted to an OD_600nm_ of 0.2 and grown till log phase (OD_600nm_ of 1) in YPD medium at 37°C with shaking at 200 rpm, followed by additional incubation of 30 min in 3 μg/ml fluconazole or the solvent control DMSO (final concentration 0.06%) with shaking at 200 rpm.

For testing the effects of proteasome inhibition with MG132 on gene expression, wild type *C*. *albicans* was grown over night in YPD medium. Overnight-grown cultures were diluted to an OD_600nm_ of 0.2 and grown in 10 ml of YPD medium to OD ~ 1.0 (approximately 3 h) at 37°C with shaking at 2000 rpm in the presence or absence of 50 μM MG132. Control cultures contained an equivalent amount of the DMSO solvent. After reaching the desired OD, one set of samples was centrifuged at 3000 rpm for 5 min and cell pellets frozen as no-drug controls. Another set of samples had 3 μg/ml of fluconazole added and treatment performed for 30 min, before centrifugation and freezing of cell pellets as above. RNA isolation and qPCRs were performed as described above.

For analysing the expression of macrophage glycolytic genes (S3D Fig), infections were performed as described above (MOI 3 *C*. *albicans*:1 macrophage), followed by using Triazol reagent to lyse macrophages with water after 1, 3, 6 and 9 h. Controls were uninfected macrophages. Macrophage RNA was isolates using Triazol, and reverse transcription and qPCRs performed as described above. *Eef2* rRNA expression was used for normalising the data.

Sequences of all qPCR primers used are listed in S2 Table.

### Fluconazole disk assay and *diskImageR* analysis

*DiskImageR* is publicly available from CRAN (the ‘Comprehensive R Archive Network’). Full instructions of the *diskImageR* pipeline are detailed in [24]. The disk diffusion plates were photographed under a consistent setting using the Phenobooth (Singer Instruments). Photograph size is standardized and the centre of the disk is located. Thirty-five millimetres radial lines are drawn every 5° starting at the disk centre and finishing at the plate edge. This is then averaged to determine the density from the disk to the edge. Variation in plating is normalized by *diskImageR*. The plate background is established by subtracting all values from the pixel intensity of a plate with clear space behind the disk. RAD_20_ values represent the distance in mm from the edge of the disk that corresponds where 20% growth reduction has occurred. FoG_20_ values are calculated using the area under the curve in slices from the disk edge to the RAD_20_ cut-off. This achieved growth is compared to the potential growth. Two-hundred μl of overnight-grown cultures were diluted to an OD_600nm_ of 0.1 (equivalent to 10^6^ cells/ml) and plated onto YPD agar using 3 mm glass beads. A single 25 μg fluconazole disk (6 mm diameter, Thermo Fisher Scientific) was placed using sterile forceps onto the middle of the plate. To ensure consistent cell density plated on each plate, the same diluted cultures were also spread on YPD agar plates without a fluconazole disk. Plates were incubated at 30°C or 37°C for 2 days and photographed.

### Microscopy

Microscopy images were taken using the EVOS FL microscope (Thermo Fisher Scientific). For S2A Fig, overnight-cultured strains were diluted to OD_600nm_ of 0.2 into prewarmed YPD medium at 37°C with shaking at 200 rpm. After 3 h, cells were harvested and vigorously vortexed to separate hyphal clumps. For S2C Fig, overnight-cultured strains were diluted to OD_600nm_ of 0.2 into prewarmed BMDM medium containing serum-free RPMI 1640 media pH 7.4 supplemented with 10 mM glucose at 37°C with shaking at 200 rpm. Images were taken at 40X magnification. Quantification of cell morphology in cultures was done in FIJI. A minimum of 100 *C*. *albicans* cells were counted for each biological replicate.

### Western blot analysis

For detecting the levels of TAP-tagged Rpn4 protein in Fig 1D, ‘tolerant’ cells from within the zone of inhibition and ‘non-tolerant’ cells outside the zone of inhibition were collected from a fluconazole disk plates grown at 37°C for 2 days and resuspended in PBS. The suspension was centrifuged at 14000 rpm for 3 min and washed with PBS in screw-cap tubes. Cell pellets were snap-frozen on dry ice and stored at -20°C awaiting protein extraction. Whole-cell protein extracts were performed by adding 100 μl glass beads, 100 μl 20% trichloroacetic acid (TCA) to cell pellets on dry ice till the TCA froze. The tubes were thawed by vortexing. Glass beads were rinsed twice in 500 μl 10% TCA and supernatant was transferred to a new tube and centrifuged at 14000 rpm for 5 min. Proteins were precipitated in 1 ml ice cold acetone and resuspended in high pH Laemmli Buffer (0.0625 M Tris pH 8.8, 2% SDS, 10% glycerol, 5% mercaptoethanol, 0.01% saturated bromophenol blue), and boiled at 100°C for 5 min. Boiled samples were loaded onto a 10% SDS-PAGE gel and transferred to a PVDF membrane at 700 mA, 300 mV using the semi-dry transfer method (Bio-Rad) for 20 min. Blocking of the membrane was performed using 5% skim milk in TBS-T (TSB with 0.1% Tween) for 1 h. For detection with the anti-TAP (Open Biosystems, Cat#CAB1991) and anti-Actin (Millipore, Cat#MAB1501) primary antibody, the membranes were incubated for 1 h at room temperature at 1:1000 dilution and 1:5000 dilution, respectively. The membranes were washed 3 times for 5 min with TBS-T followed by incubation with HRP-conjugated secondary antibodies (Sigma-Aldrich, anti-TAP secondary antibody Cat#A0504 and anti-Actin secondary antibody Cat#A4416) at room temperature at 1:1000 dilution in 5% skim milk in TBS-T for 1 h. Membranes were visualized using Clarity Western ECL substrate (Bio-Rad) and exposed to medical X-ray film [48].

Testing the effects of fluconazole on the accumulation of ubiquitinated proteins: cultures of wild type and *rpn4Δ/Δ C*. *albicans* were grown overnight in YPD medium. Overnight-grown cultures were diluted to an OD_600nm_ of 0.2 and grown in 10 ml of YPD medium for 2 h at 37°C with shaking at 200 rpm. After that, the no-drug samples were harvested by centrifugation at 3000 rpm for 5 min, and cell pellets snap frozen. The fluconazole samples were obtained by adding 3ug/ml fluconazole and incubating for 4 h before harvesting cells as above. Protein extracts were prepared using the TCA method as above and separated on a 10% SDS PAGE gel. Western blots were performed using the anti-Ubiquitin antibody from Santa Cruz Biotechnologies (1: 2000 dilution, 1 h incubation at room temperature), followed by the anti-mouse HRP secondary antibody (1: 10000 dilution at room temperature). For experiments addressing the effects of MG132 on proteasome inhibition, overnight fungal cultures were diluted to an OD_600nm_ of 0.2 and grown in 10 ml of YPD medium to OD ~ 1.0 (approximately 3 h) at 37°C with shaking at 200 rpm in the presence or absence of 50 μM MG132. Controls contained an equivalent amount of the DMSO solvent. Cells were harvested and prepared for Western blots as above.

### Antifungal susceptibility tests

Minimal inhibitory concentrations (MICs) of fluconazole were determined using the broth microdilution method according to CLSI guideline M27-A3. Single colonies of freshly streaked *C*. *albicans* cells (< three days old) were scrapped off a YPD plate and diluted to 2 x 10^3^ cells/ml in YPD medium. Fluconazole drug concentrations ranged from 0.25 μg/ml to 128 μg/ml. One-hundred-microliter of 2-fold serial dilution of the drugs were added into wells of 96-well plates containing 100 μl of *C*. *albicans*. A plate reader (Tecan) was used to determine optical density at OD_600nm_ after 24 h and 48 h. MIC was defined as the lowest concentration resulting in no fungal growth. MIC_80_ was defined as the concentration resulting in an inhibition of at least 80% of fungal growth. For analysis of susceptibility to various stressors, ten-fold serial dilutions of overnight-grown cultures starting from an optical density (OD_600nm_) of 0.5 were plated on control plates or plates supplemented with various compounds as indicated in the figure legends. Plates were incubated at 30°C or 37°C for 2 days and then photographed.

### RNA-seq analysis

Wild type and *rpn4Δ/Δ* cells from overnight-grown cultures were diluted to an OD_600nm_ of 0.2 and grown in 20 ml of YPD medium at 37°C with shaking at 200 rpm till log phase (OD_600nm_ of 1). Log phase cultures were treated with 3 μg/ml fluconazole or the solvent control DMSO (final concentration 0.06%) for 30 min at 37°C with shaking at 200 rpm. Cultures (10 ml) were then centrifuged at 3000 rpm at 4°C for 3 min, washed with water and pelleted into screw-cap tubes as ‘+FLC’ or ‘-FLC’ samples. Cell pellets were snap-frozen on dry ice and stored at -80°C awaiting RNA extraction.

Total RNA from three independent biological repeats were extracted using the hot phenol method as we described previously [46]. Quant-seq libraries were sequenced with the Illumina Hiseq1500 platform according to the manufacturers’ instructions. The raw sequencing data were processed as described previously [46,49]. The cut-off for genes to be included in the analysis was ≥ 5 counts per million (CPM) in at least 2 samples. The RNA-seq dataset can be viewed at https://degust.erc.monash.edu/degust/compare.html?code=7626de961da6ddac4911adffc3b6e7ca#/. The volcano plots in Fig 2A were constructed in GraphPad Prism 8 with no FDR cut-off, log2 of 0 (version 8.4.3). The heatmap in Figs 2B and 3C representing changes in differential gene expression were constructed using the online software Morpheus (https://software.broadinstitute.org/morpheus, FDR < 0.05, log2 of 0.585 i.e., ≥ 1.5 fold change). The multidimensional scaling (MDS) plot in S6C Fig was obtained from the Degust session above (no cut-off applied). The heatmap in S6D Fig representing expression of genes relative to the average of gene expression across all samples was obtained from the Degust session above (FDR < 0.05). The Venn diagram in Fig 2C shows the overlap between genes differentially expression in wild type and *rpn4Δ/Δ* cells by fluconazole (FDR < 0.05, log2 of 0.585, i.e. ≥ 1.5 fold change). The gene ontology (GO) term analysis of biological process in differentially expressed genes was performed using the tools at the Candida Genome Database.

Genome sequencing was performed on wild type (YCAT 641, SN425), *rpn4Δ/Δ* clone x (YCAT 1099) and *rpn4Δ/Δ* clone y (YCAT 1100) strains (S1 Table). Strains were grown in standard culture conditions (growth for 14 h in YPD medium 30°C) and genomic DNA was extracted via the phenol/chloroform/isoamyl alcohol (25:24:1) method. Whole genome sequencing (WGS) was performed by Deakin Genomics Centre on a MiSeq V3 machine. For the three strains, WGS libraries were prepared with Illumina DNA Prep with unique dual indexing, using an insert size of 300bp. To check for evidence of changes to chromosome copy number the WGS paired-end fastq files were uploaded to the YMAP analysis site [50]. The reference genome selected was SC5314 (ver. A21-s02-m08-r09) with the wild type strain (YCAT 641) selected as the parental strain for analysing *rpn4Δ/Δ* clone x and *rpn4Δ/Δ* clone y strains. The results of this analysis are shown in S5 Fig. Further to this, the RNA-seq data was analysed for evidence of chromosomal bias in gene expression. The sequence data was processed as described above for RNA-seq to produce gene-level counts per sample. These counts were TMM normalised [51] and converted to log CPM values before fitting a voom/limma [52] linear model to estimate log fold change per gene shown in boxplots in S6A and S6B Fig. A normal distribution per chromosome was fitted to these producing an estimated log fold change per chromosome which was used to test whether the chromosome showed evidence of biased gene expression.

### Statistical analysis

GraphPad Prism was used to perform statistical analyses. Details of statistical method used are described in the figure legends. The number of biological replicates and the statistical values for the individual experiments are also stated in the figure legends.

## Supporting information

S1 Fig Stress response phenotypes of the *rpn4Δ/Δ* mutant. **A.** Wild type (WT), *rpn4Δ/Δ* and complemented strains were grown on YPD plates containing the indicated compounds. Plates were incubated at 30 or 37°C for 2 days and photographed. The solvent control plate for amphotericin B, fluconazole and antimycin A is shown next to the drug plates. For all other stressors, the control plate is YPD (top of panel). Three independent experiments were performed and gave equivalent results. One representative experiment is shown here. **B.** Growth rates of wild type (WT), *rpn4Δ/Δ* and complemented strains in tissue culture (RPMI-based) medium at 37°C. The medium is the same as used for macrophage infections (see Materials and Methods). Growth was assessed by measuring OD_600nm_ over a period of 20 h. Shown are the mean values of three independent experiments, each independent experiment was analysed in two technical replicates. Error bars represent the standard error of mean. **C.** Calculations of growth parameters (growth rate, doubling time, carrying capacity) based on data in panel B. The calculations were performed using the R package Growthcurver [53]. Data points are from three independent experiments. Horizontal bars represent the mean and error bars represent the standard error of mean. * P < 0.05 (2-way ANOVA Bonferroni’s multiple comparison test). Only significant statistical comparisons are shown.
(TIF)

S2 Fig Morphogenesis of the *rpn4Δ/Δ* mutant. **A.** Fungal morphology of wild type (WT), *rpn4Δ/Δ* and complemented strains in YPD media after 3 h of growth at 30°C. Five independent experiments were performed and gave equivalent results. One representative experiment is shown. **B.** Percentage of different cell morphologies (yeast, pseudohyphae, hyphae) relative to the total number of cells based on experiments described in panel A. Data points are from five independent experiments. Horizontal bars represent the mean and error bars represent the standard error of mean. *** P < 0.001 (2-way ANOVA Bonferroni’s multiple comparison test). Only significant statistical comparisons are shown. **C.** Fungal morphology of WT, *rpn4Δ/Δ* and complemented strains in macrophage infection medium after 3 h of growth at 37°C. Three independent experiments were performed and gave equivalent results. One representative experiment is shown. Scale bar is 20 μm. **D.** Representative images from the live cell microscopy at 4.5 h post-infection of macrophages with wild type, *rpn4Δ/Δ* and complemented strains. Scale bar is 30 μm.
(TIF)

S3 Fig *C*. *albicans* Rpn4 promotes macrophage killing. **A.** Microscopy images and phagocytic index counts of mouse bone marrow-derived macrophages (BMDMs) after infection with *C*. *albicans* wild type (WT) *rpn4Δ/Δ* deletion clones x and y, and their respective complemented strains. The MOI was 2 *Candida*: 1 macrophage. The DAPI channel was used to image calcofluor white stained cells. The image has been falsely coloured in cyan to improve visibility. **B.** Live cell imaging measuring the death of BMDMs after infection with *C*. *albicans* WT *rpn4Δ/Δ* deletion clones x and y, and their respective complemented strains. The multiplicity of infection (MOI) was 1.5 *Candida*:1 macrophage. Three independent experiments were performed and are shown here separately (mean values of two technical repeats with the error bars that represent the standard error of mean). **C.** Glucose depletion in the medium during BMDM infection with the indicated strains (MOI 1.5 *Candida*:1 macrophage). Data points are from four independent experiments. Shown are the averages and the standard error of the mean. ** P < 0.01; *** P < 0.001 (2-way ANOVA Bonferroni’s multiple comparison test). Only significant statistical comparisons are shown. **D.** qPCR for of the indicated macrophage metabolic genes upon infection with *C*. *albicans* WT, *rpn4Δ/Δ* or left uninfected (MOI 3 *Candida*:1 macrophage). Data points are from three independent experiments. Horizontal bars represent the mean and error bars represent the standard error of mean. * p<0.05; **P < 0.01; ***P < 0.001; **** P < 0.0001 (2-way ANOVA with Tukey or Bonferroni’s multiple comparison test). Only significant statistical comparisons are shown.
(TIF)

S4 Fig Uncropped Western blots of Rpn4-TAP. Uncropped Westerns shown in Fig 1D.
(TIF)

S5 Fig Analysis of genome sequencing data for *rpn4Δ/Δ* mutant clones. YMAP was used to depict chromosome in wild type (SN425) and the two *rpn4Δ/Δ* clones (x and y) (CNV standard view, figures generated using http://lovelace.cs.umn.edu/Ymap/). Copy number variations per position are displayed as black histograms along the length of each chromosome. The y-axis represents the relative chromosome copy numbers, based on the whole genome ploidy. The numbers to the right of each chromosome are copy number calculations.
(TIF)

S6 Fig Analysis of the RNA-seq data for the two *rpn4Δ/Δ* clones. **A.** Box plots representing differentially regulated genes in *rpn4Δ/Δ* mutants by chromosome. The top panel represents log fold change (> 1.5 and < 0.5) in gene expression across the chromosome for untreated (DMSO) *rpn4Δ/Δ* clone x and *rpn4Δ/Δ* clone y, relative to the untreated (DMSO) wild type strain. The bottom panel represents log fold change (> 1.5 and < 0.5) in gene expression across the chromosome for fluconazole-treated (FLC) *rpn4Δ/Δ* clone x and *rpn4Δ/Δ* clone y, relative to fluconazole-treated (FLC) wild type strain. The x-axis represents the 8 chromosomes of *C*. *albicans*. The black line within the box represents median gene expression, and the box shows the inter-quartile range (IRQ) with the whiskers extending 1.5*IQR. **B.** Box plots representing differentially regulated genes in untreated versus fluconazole-treated *rpn4Δ/Δ* mutants. The left panel represents log fold change (> 1.5 and < 0.5) in gene expression across the chromosome for fluconazole-treatment (FLC) relative to the untreated (DMSO) for *rpn4Δ/Δ* clone x. The left panel represents log fold change (> 1.5 and < 0.5) in gene expression across the chromosome for fluconazole-treatment (FLC) relative to the untreated (DMSO) for *rpn4Δ/Δ* clone y. **C.** An MDS plot of the RNA-seq samples. Calculation of percentage variance between Dimension 1 and Dimension 2 from the MDS plot (no cut-off applied). **D.** Heat maps of differentially regulated genes in wild type untreated cells, wild type FLC -treated cells, *rpn4Δ/Δ* clone x and *rpn4Δ/Δ* clone y FLC-treated cells, and *rpn4Δ/Δ* clone x and *rpn4Δ/Δ* clone y untreated cells, FDR < 0.05).
(TIF)

S7 Fig Growth conditions for the RNA-seq experiment. Colony forming units (CFU/ml) of wild type (WT), *rpn4Δ/Δ* and complemented strains supplemented with 3 μg/ml fluconazole or matched DMSO controls (final concentration 0.06%). Cell were treated for 30 min and then diluted and plated on YPD agar plates. The growth temperature was 37°C. CFUs were determined after 2 days. **, P < 0.01; **, P < 0.001 (2-way ANOVA Bonferroni’s multiple comparison test). Only significant statistical comparisons are shown.
(TIF)

S8 Fig Fluconazole susceptibility of the ubiquitin mutant *ubi4Δ/Δ*. A. Fluconazole disk diffusion assays were performed with 25 μg fluconazole for wild type (WT) and *ubi4Δ/Δ* strains at 37°C. Three independent experiments were performed and gave equivalent results. One representative experiment is shown. The top panel (without the disk) shows untreated conditions (no drug). B. *DiskImageR* analysis of RAD_20_ and FoG_20_ values on experiments according to panel A. Data points represent three independent experiments, horizontal bars represent the mean and error bars represent the standard error of mean. * P < 0.05; ** P < 0.01; n.s. not significant (2-way ANOVA Bonferroni’s multiple comparison test).
(TIF)

S1 Dataset RNA-seq data analysis. Gene Ontology (GO) analyses were performed with the tools at the Candida Genome database.
(XLSX)

S2 Dataset Bioinformatic analyses of putative Rpn4 gene targets in *C*. *albicans*. (XLSX)

S3 Dataset Data underlying the graphs presented in the figures. (XLSX)

S1 Table Strains used in this study. (XLSX)

S2 Table Primers used in this study. (XLSX)

S1 Movie Live cell imaging of infected macrophages. Murine bone marrow-derived macrophages were infected with *C*. *albicans* (wild type, *rpn4Δ/Δ* clones x and y and their respective complemented strains. Hyphal formation in macrophages was viewed with brightfield live cell microscopy from 2–9 h post-infection. The scale bar represents 100 μm. Clips were generated using FIJI; brightness and contrast maxima were decreased to 12 000–15 000.
(MP4)
