# Supplementary figures and images for: The proteasome regulator Rpn4 controls antifungal drug tolerance by coupling protein homeostasis with metabolic responses to drug stress
Source: PLoS Pathog. 2023 Apr 19;19(4):e1011338. doi: 10.1371/journal.ppat.1011338 (PMC10150987; doi:10.1371/journal.ppat.1011338)

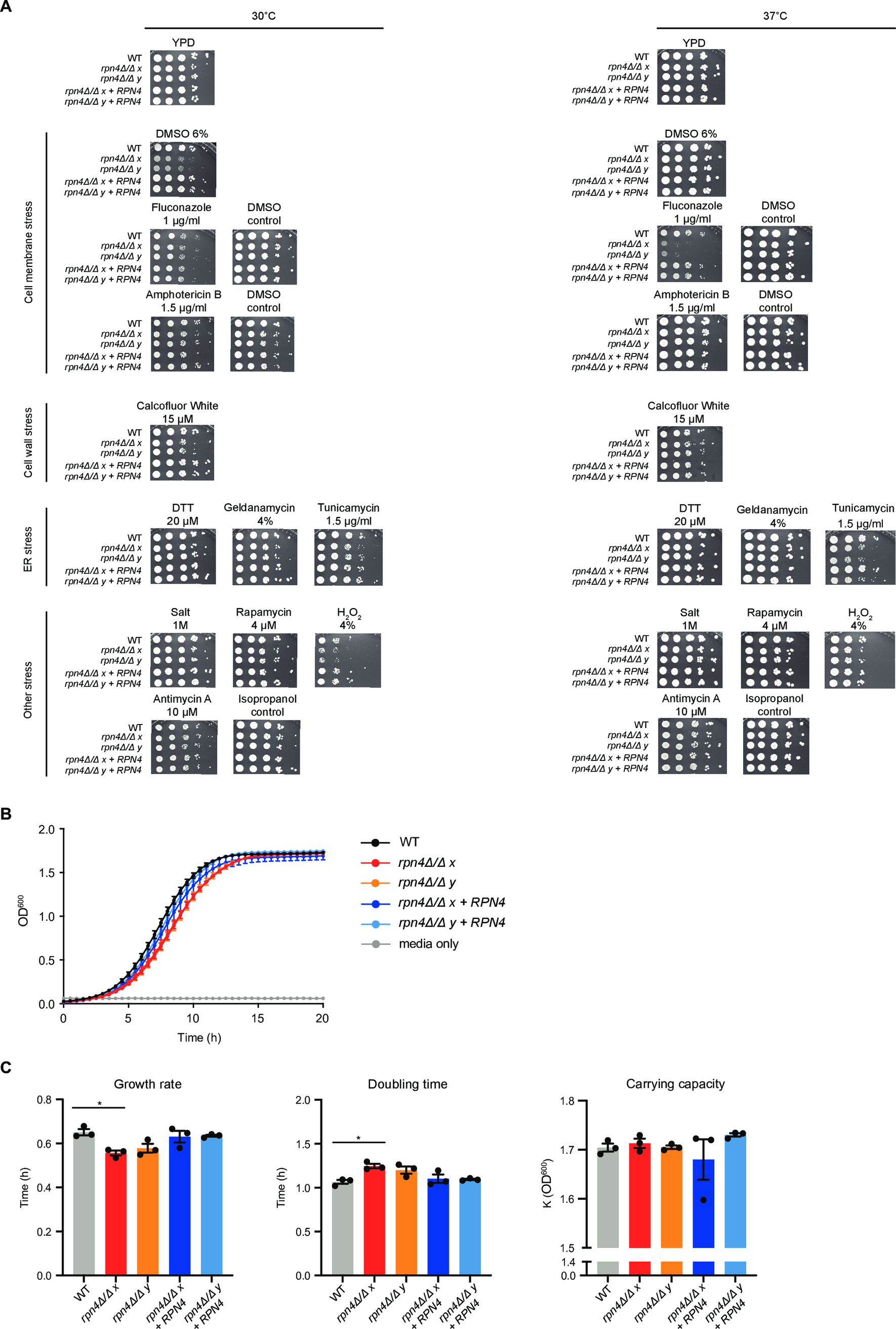

Supplement: S1 Fig — A. Wild type (WT), rpn4Δ/Δ and complemented strains were grown on YPD plates containing the indicated compounds. Plates were incubated at 30 or 37°C for 2 days and photographed. The solvent control plate for amphotericin B, fluconazole and antimycin A is shown next to the drug plates. For all other stressors, the control plate is YPD (top of panel). Three independent experiments were performed and gave equivalent results. One representative experiment is shown here. B. Growth rates of wild type (WT), rpn4Δ/Δ and complemented strains in tissue culture (RPMI-based) medium at 37°C. The medium is the same as used for macrophage infections (see Materials and Methods). Growth was assessed by measuring OD600nm over a period of 20 h. Shown are the mean values of three independent experiments, each independent experiment was analysed in two technical replicates. Error bars represent the standard error of mean. C. Calculations of growth parameters (growth rate, doubling time, carrying capacity) based on data in panel B. The calculations were performed using the R package Growthcurver [53]. Data points are from three independent experiments. Horizontal bars represent the mean and error bars represent the standard error of mean. * P < 0.05 (2-way ANOVA Bonferroni’s multiple comparison test). Only significant statistical comparisons are shown. (TIF) [file ppat.1011338.s001.tif]

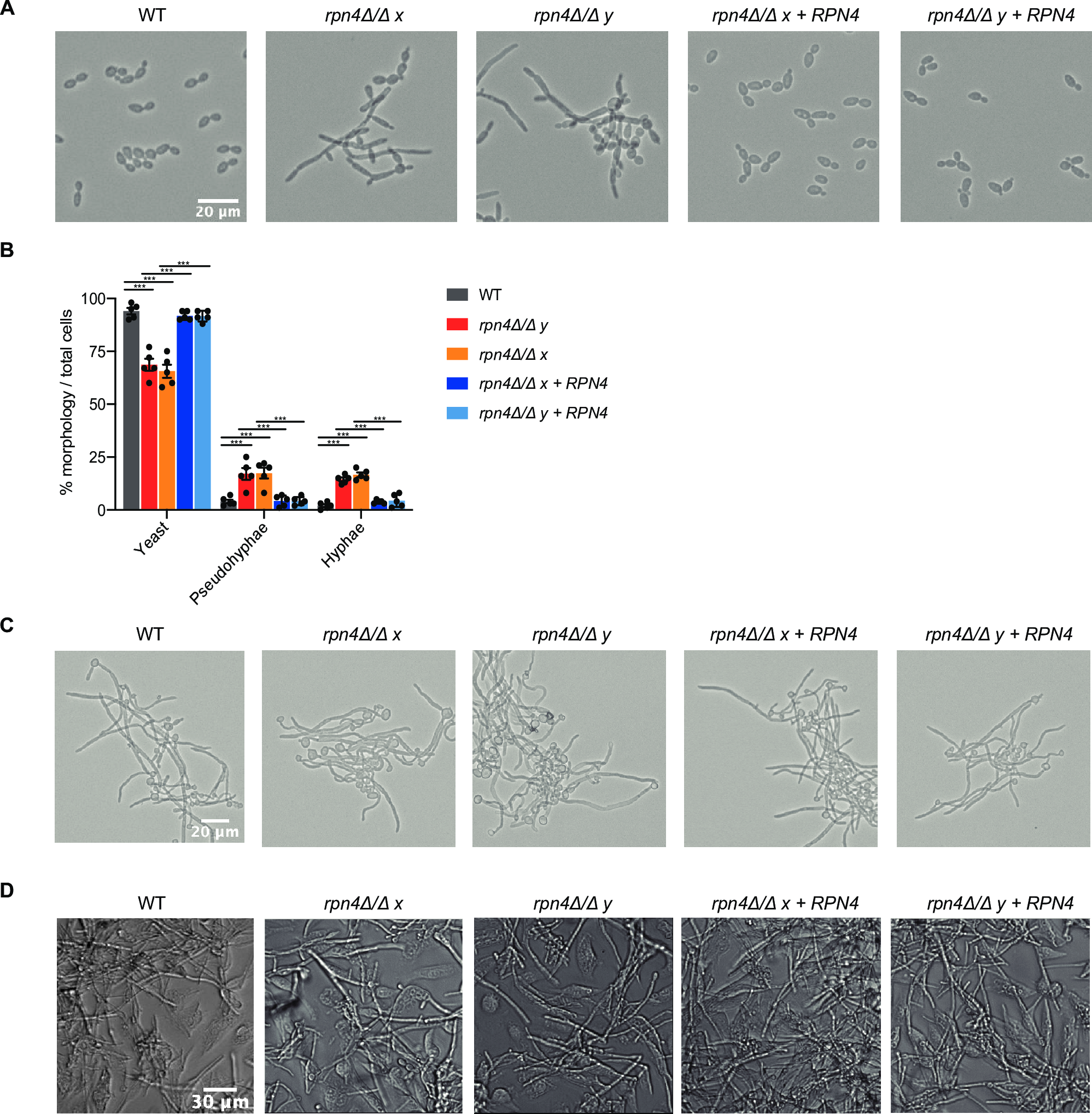

Supplement: S2 Fig — A. Fungal morphology of wild type (WT), rpn4Δ/Δ and complemented strains in YPD media after 3 h of growth at 30°C. Five independent experiments were performed and gave equivalent results. One representative experiment is shown. B. Percentage of different cell morphologies (yeast, pseudohyphae, hyphae) relative to the total number of cells based on experiments described in panel A. Data points are from five independent experiments. Horizontal bars represent the mean and error bars represent the standard error of mean. *** P < 0.001 (2-way ANOVA Bonferroni’s multiple comparison test). Only significant statistical comparisons are shown. C. Fungal morphology of WT, rpn4Δ/Δ and complemented strains in macrophage infection medium after 3 h of growth at 37°C. Three independent experiments were performed and gave equivalent results. One representative experiment is shown. Scale bar is 20 μm. D. Representative images from the live cell microscopy at 4.5 h post-infection of macrophages with wild type, rpn4Δ/Δ and complemented strains. Scale bar is 30 μm. (TIF) [file ppat.1011338.s002.tif]

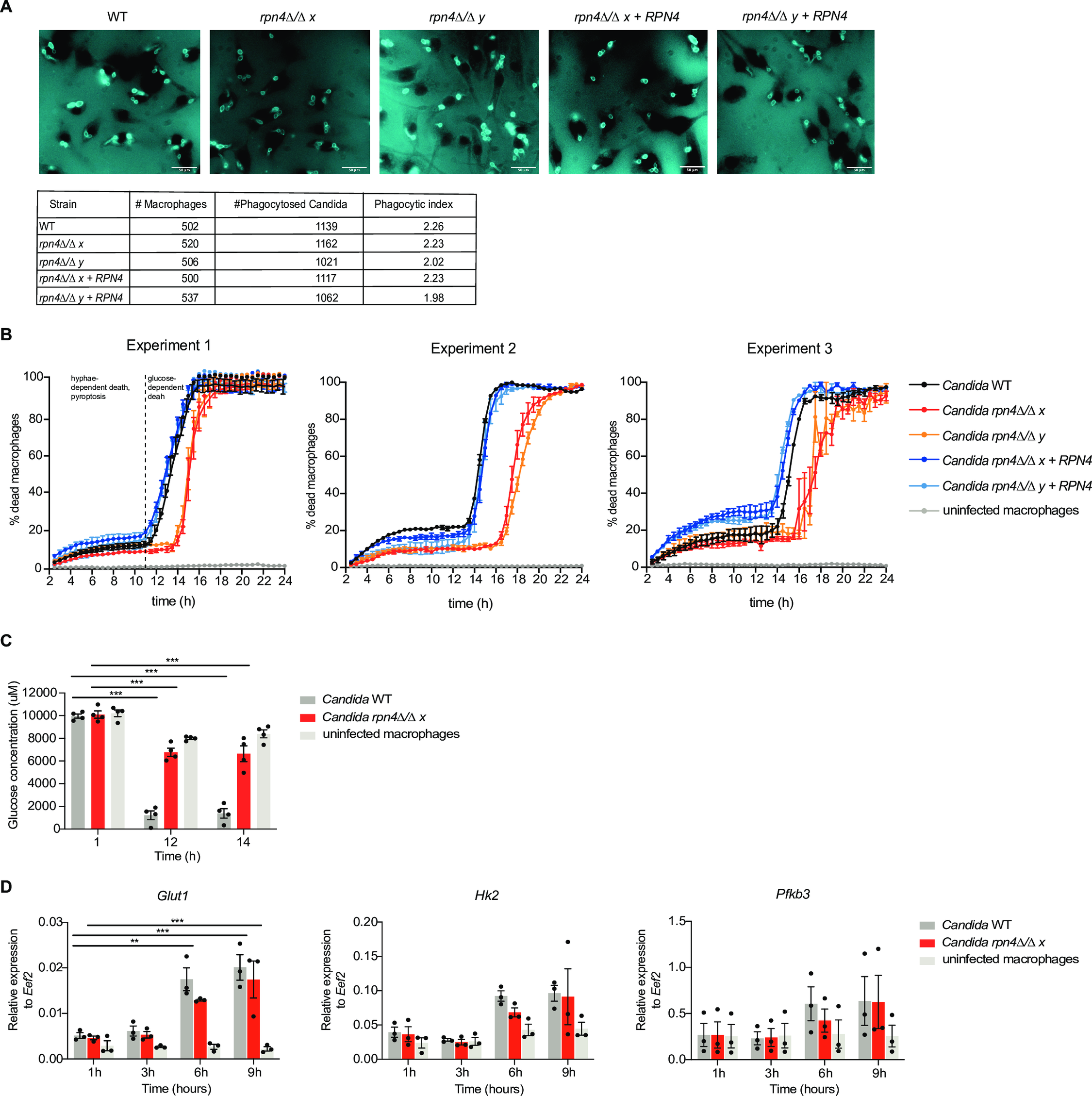

Supplement: S3 Fig — A. Microscopy images and phagocytic index counts of mouse bone marrow-derived macrophages (BMDMs) after infection with C. albicans wild type (WT) rpn4Δ/Δ deletion clones x and y, and their respective complemented strains. The MOI was 2 Candida: 1 macrophage. The DAPI channel was used to image calcofluor white stained cells. The image has been falsely coloured in cyan to improve visibility. B. Live cell imaging measuring the death of BMDMs after infection with C. albicans WT rpn4Δ/Δ deletion clones x and y, and their respective complemented strains. The multiplicity of infection (MOI) was 1.5 Candida:1 macrophage. Three independent experiments were performed and are shown here separately (mean values of two technical repeats with the error bars that represent the standard error of mean). C. Glucose depletion in the medium during BMDM infection with the indicated strains (MOI 1.5 Candida:1 macrophage). Data points are from four independent experiments. Shown are the averages and the standard error of the mean. ** P < 0.01; *** P < 0.001 (2-way ANOVA Bonferroni’s multiple comparison test). Only significant statistical comparisons are shown. D. qPCR for of the indicated macrophage metabolic genes upon infection with C. albicans WT, rpn4Δ/Δ or left uninfected (MOI 3 Candida:1 macrophage). Data points are from three independent experiments. Horizontal bars represent the mean and error bars represent the standard error of mean. * p<0.05; **P < 0.01; ***P < 0.001; **** P < 0.0001 (2-way ANOVA with Tukey or Bonferroni’s multiple comparison test). Only significant statistical comparisons are shown. (TIF) [file ppat.1011338.s003.tif]

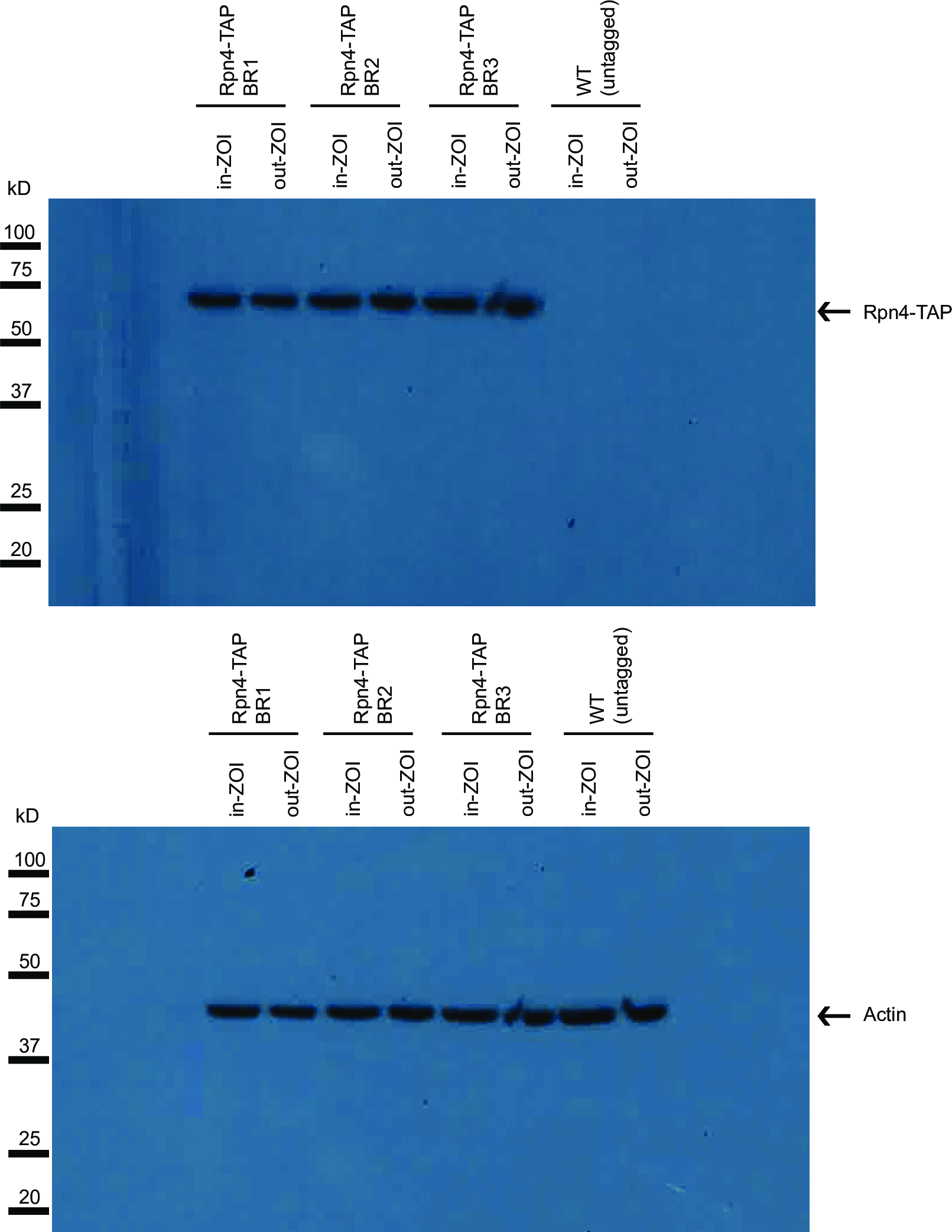

Supplement: S4 Fig — Uncropped Westerns shown in Fig 1D. (TIF) [file ppat.1011338.s004.tif]

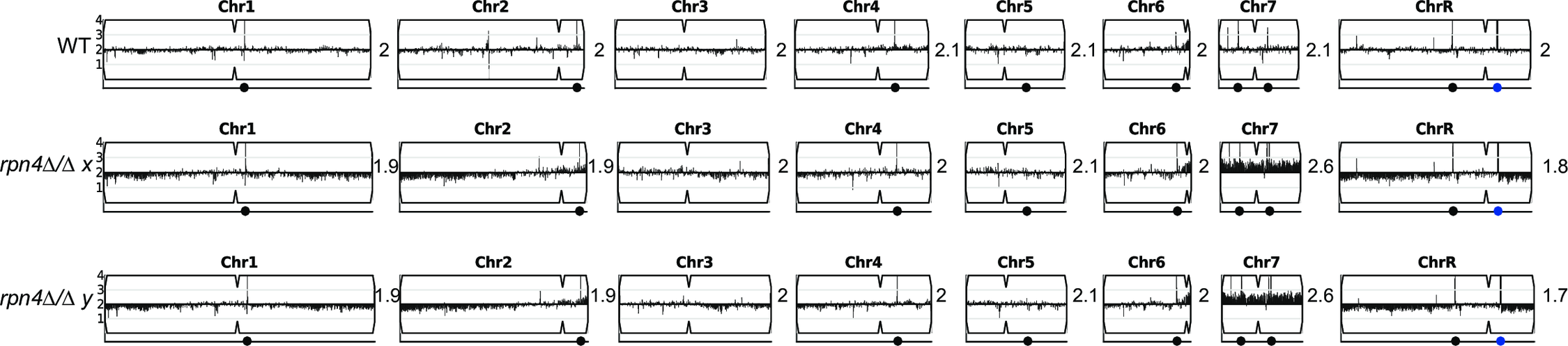

Supplement: S5 Fig — YMAP was used to depict chromosome in wild type (SN425) and the two rpn4Δ/Δ clones (x and y) (CNV standard view, figures generated using http://lovelace.cs.umn.edu/Ymap/). Copy number variations per position are displayed as black histograms along the length of each chromosome. The y-axis represents the relative chromosome copy numbers, based on the whole genome ploidy. The numbers to the right of each chromosome are copy number calculations. (TIF) [file ppat.1011338.s005.tif]

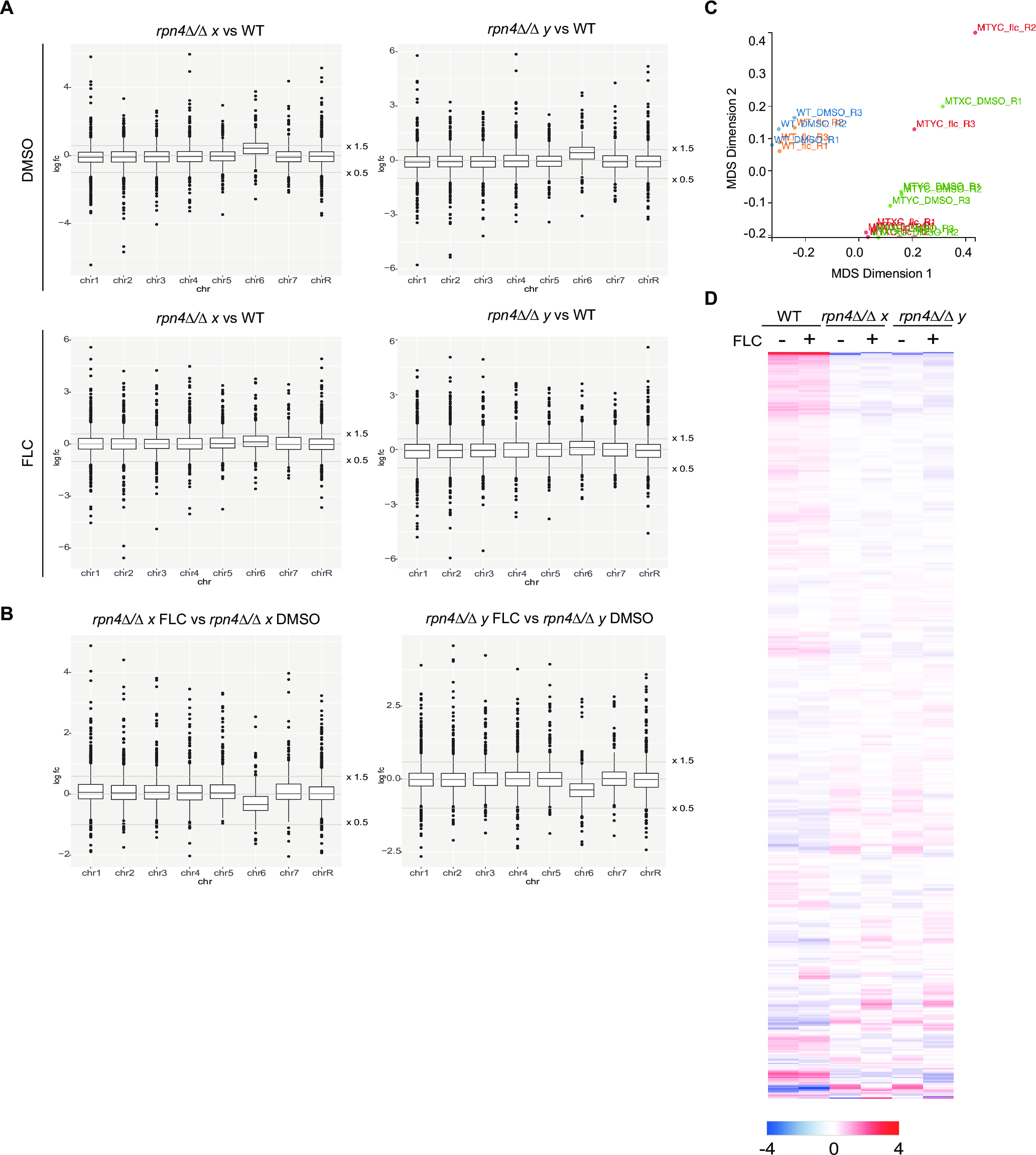

Supplement: S6 Fig — A. Box plots representing differentially regulated genes in rpn4Δ/Δ mutants by chromosome. The top panel represents log fold change (> 1.5 and < 0.5) in gene expression across the chromosome for untreated (DMSO) rpn4Δ/Δ clone x and rpn4Δ/Δ clone y, relative to the untreated (DMSO) wild type strain. The bottom panel represents log fold change (> 1.5 and < 0.5) in gene expression across the chromosome for fluconazole-treated (FLC) rpn4Δ/Δ clone x and rpn4Δ/Δ clone y, relative to fluconazole-treated (FLC) wild type strain. The x-axis represents the 8 chromosomes of C. albicans. The black line within the box represents median gene expression, and the box shows the inter-quartile range (IRQ) with the whiskers extending 1.5*IQR. B. Box plots representing differentially regulated genes in untreated versus fluconazole-treated rpn4Δ/Δ mutants. The left panel represents log fold change (> 1.5 and < 0.5) in gene expression across the chromosome for fluconazole-treatment (FLC) relative to the untreated (DMSO) for rpn4Δ/Δ clone x. The left panel represents log fold change (> 1.5 and < 0.5) in gene expression across the chromosome for fluconazole-treatment (FLC) relative to the untreated (DMSO) for rpn4Δ/Δ clone y. C. An MDS plot of the RNA-seq samples. Calculation of percentage variance between Dimension 1 and Dimension 2 from the MDS plot (no cut-off applied). D. Heat maps of differentially regulated genes in wild type untreated cells, wild type FLC -treated cells, rpn4Δ/Δ clone x and rpn4Δ/Δ clone y FLC-treated cells, and rpn4Δ/Δ clone x and rpn4Δ/Δ clone y untreated cells, FDR < 0.05). (TIF) [file ppat.1011338.s006.tif]

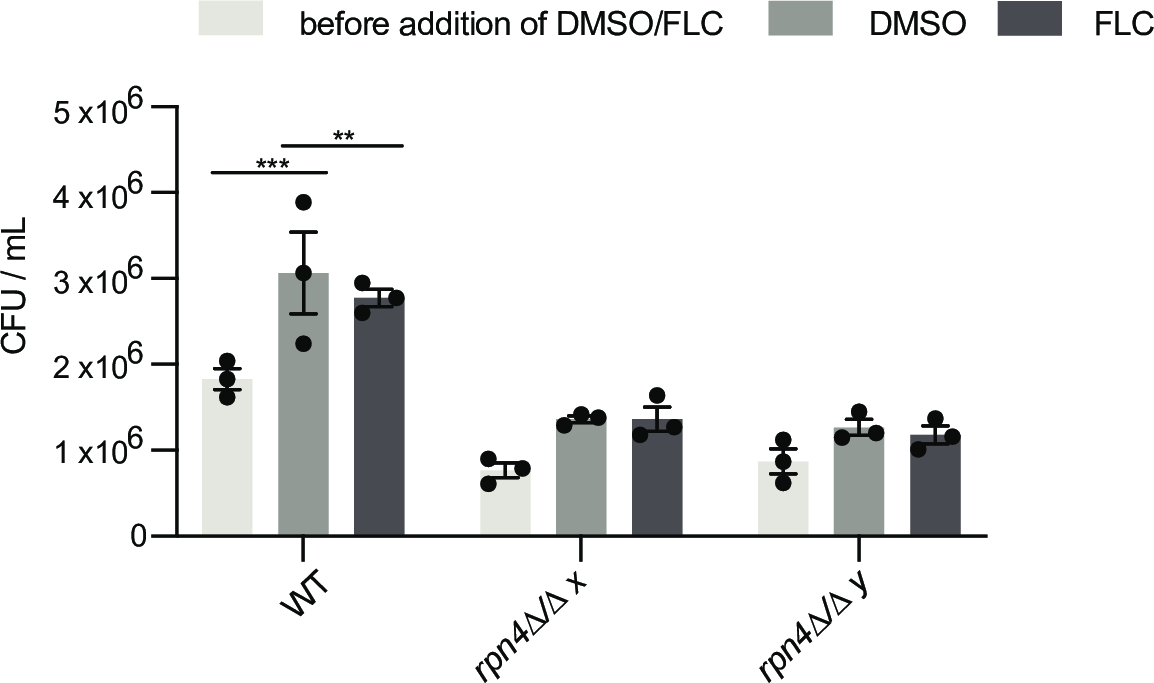

Supplement: S7 Fig — Colony forming units (CFU/ml) of wild type (WT), rpn4Δ/Δ and complemented strains supplemented with 3 μg/ml fluconazole or matched DMSO controls (final concentration 0.06%). Cell were treated for 30 min and then diluted and plated on YPD agar plates. The growth temperature was 37°C. CFUs were determined after 2 days. **, P < 0.01; **, P < 0.001 (2-way ANOVA Bonferroni’s multiple comparison test). Only significant statistical comparisons are shown. (TIF) [file ppat.1011338.s007.tif]

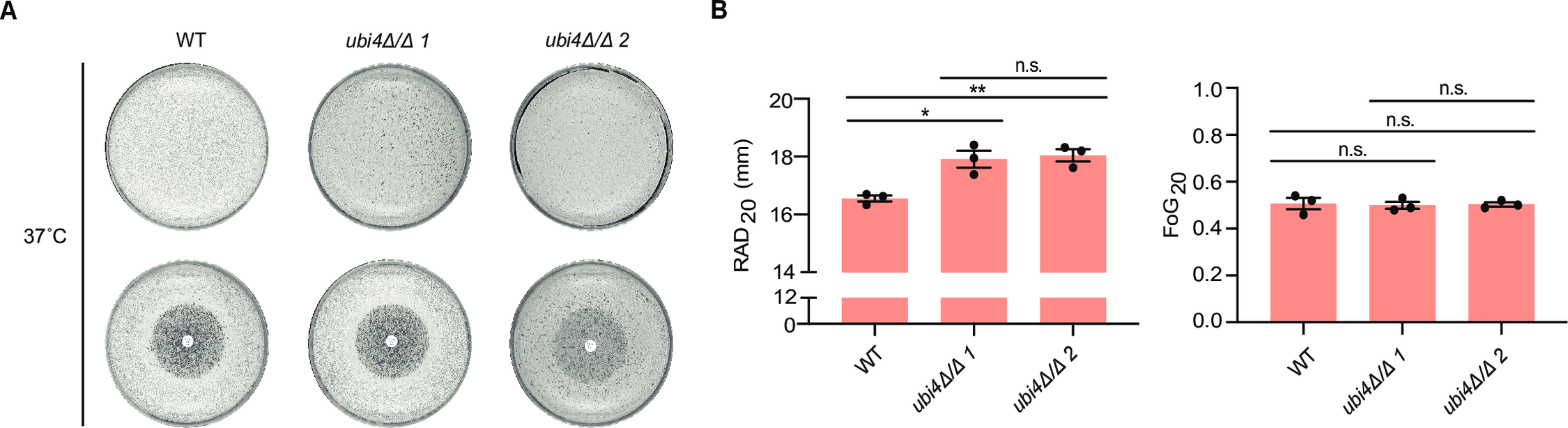

Supplement: S8 Fig — A. Fluconazole disk diffusion assays were performed with 25 μg fluconazole for wild type (WT) and ubi4Δ/Δ strains at 37°C. Three independent experiments were performed and gave equivalent results. One representative experiment is shown. The top panel (without the disk) shows untreated conditions (no drug). B. DiskImageR analysis of RAD20 and FoG20 values on experiments according to panel A. Data points represent three independent experiments, horizontal bars represent the mean and error bars represent the standard error of mean. * P < 0.05; ** P < 0.01; n.s. not significant (2-way ANOVA Bonferroni’s multiple comparison test). (TIF) [file ppat.1011338.s008.tif]
